# Navigating Past Oceans: Comparing Metabarcoding and Metagenomics of Marine Ancient Sediment Environmental DNA

**DOI:** 10.1111/1755-0998.14086

**Published:** 2025-02-20

**Authors:** Luke E. Holman, Giulia Zampirolo, Richard Gyllencreutz, James Scourse, Tobias Frøslev, Christian Carøe, Shyam Gopalakrishnan, Mikkel Winther Pedersen, Kristine Bohmann

**Affiliations:** ^1^ Section for Molecular Ecology and Evolution, Globe Institute University of Copenhagen Copenhagen Denmark; ^2^ Department of Geological Sciences Stockholm University Stockholm Sweden; ^3^ Bolin Centre for Climate Research Stockholm University Stockholm Sweden; ^4^ Centre for Geography and Environmental Science University of Exeter Exeter UK; ^5^ Centre for Ancient Environmental Genomics, Globe Institute University of Copenhagen Copenhagen Denmark; ^6^ Global Biodiversity Information Facility Copenhagen Denmark; ^7^ Novonesis A/S Lyngby Denmark; ^8^ Centre for Hologenomics, Globe Institute University of Copenhagen Copenhagen Denmark

**Keywords:** 18S ribosomal RNA, ancient eDNA, ancient oceans, marine biodiversity, sedaDNA

## Abstract

The condition of ancient marine ecosystems provides context for contemporary biodiversity changes in human‐impacted oceans. Sequencing sedimentary ancient DNA (sedaDNA) is an emerging method for generating high‐resolution biodiversity time‐series data, offering insights into past ecosystems. However, few studies directly compare the two predominant sedaDNA sequencing approaches: metabarcoding and shotgun‐metagenomics, and it remains unclear if these methodological differences affect diversity metrics. We compared these methods using sedaDNA from an archived marine sediment record sampled in the Skagerrak, North Sea, spanning almost 8000 years. We performed metabarcoding of a eukaryotic 18S rRNA region (V9) and sequenced 153–229 million metagenomic reads per sample. Our results show limited overlap between metabarcoding and metagenomics, with only three metazoan genera detected by both methods. For overlapping taxa, metabarcoding detections became inconsistent for samples older than 2000 years, while metagenomics detected taxa throughout the time series. We observed divergent patterns of alpha diversity, with metagenomics indicating decreased richness towards the present and metabarcoding showing an increase. However, beta diversity patterns were similar between methods, with discrepancies only in metazoan data comparisons. Our findings demonstrate that the choice of sequencing method significantly impacts detected biodiversity in an ancient marine sediment record. While we stress that studies with limited variation in DNA degradation among samples may not be strongly affected, researchers should exonerate methodological explanations for observed biodiversity changes in marine sediment cores, particularly when considering alpha diversity, before making ecological interpretations.

## 1 Introduction

Sedimentary ancient DNA (sedaDNA) is increasingly used to reconstruct past biodiversity (Capo et al. 2021; Nguyen et al. 2023). Through high‐throughput sequencing of ancient environmental DNA fragments preserved in sedimentary records, such as sediment cores from lakes or marine ecosystems, it is possible to reconstruct time series data for a wide range of taxa over substantial periods of time (Bálint et al. 2018; Kjær et al. 2022). This has allowed researchers to describe ancient shifts in marine biodiversity (Armbrecht et al. 2022), observe changes in marine species composition in response to abiotic changes (Zimmermann et al. 2023) and characterise ecosystems with no modern analogue (Kjær et al. 2022). There are two DNA‐based methods typically used to analyse ancient sedimentary environmental DNA; metabarcoding, in which a homologous DNA fragment (barcode) is amplified across a group of taxa and sequenced (Taberlet et al. 2012); and shotgun‐metagenomics (hereafter metagenomics) where the total DNA pool is sequenced. At present, metabarcoding is the most commonly used approach in sedaDNA research, with lab protocols, primer sets and bioinformatic software reaching a more mature and standardised state compared to metagenomics (Der Sarkissian et al. 2021; Heintzman et al. 2023; Holman et al. 2023; Revéret et al. 2023). Despite the rapid increase in sedaDNA publications (Capo et al. 2021; Nguyen et al. 2023), only a few sedaDNA studies have directly compared metabarcoding and metagenomics to describe past biodiversity (Armbrecht et al. 2021; Murchie et al. 2020; Wang et al. 2021). Moreover, as most sedaDNA studies use only a single technique, a full understanding of any differences between methods is critical to derive ecological inferences from metabarcoding or metagenomic data.

In contrast to work in sedaDNA, many studies have compared metabarcoding and metagenomics in contemporary environmental material, such as soil, seawater, freshwater, and faecal samples (e.g. Bista et al. 2018; Stat et al. 2017; Tessler et al. 2017). Together, these studies show that, in comparison to metagenomics, metabarcoding yields a greater proportion of sequences generated that can be taxonomically identified (Jovel et al. 2016; Stat et al. 2017; Tessler et al. 2017). This is expected given the limited resolution of some regions of the genome for taxonomic assignment and the currently incomplete genomic reference databases (Breitwieser et al. 2019; Huson et al. 2016; Lewin et al. 2018). Metabarcoding and metagenomics often detect different taxa, with only a small minority of observations overlapping (Latz et al. 2022; Tessler et al. 2017). Due to primer biases and PCR stochasticity metabarcoding amplifies only a subset of the total diversity, thus producing an informative but skewed view of total biodiversity. In contrast, in metagenomics the total DNA pool is sequenced and thus the portion of the community with the greatest proportion of taxonomically informative DNA is detected. There are also differences in the relative abundance of sequence assigned to detected taxa between metabarcoding and metagenomics, which can be attributed to metabarcoding primer bias distorting the observed read proportions (Bista et al. 2018; Clooney et al. 2016; Jovel et al. 2016; Latz et al. 2022; Machida et al. 2021; Tessler et al. 2017). Only a few comparisons have been made between diversity metrics produced using metabarcoding and metagenomics of modern samples. Here, some studies have found greater alpha diversity in metagenomics compared to metabarcoding (Logares et al. 2014), while others have found the opposite trend with greater alpha diversity using metabarcoding (Clooney et al. 2016; Tessler et al. 2017). Similarly, studies comparing beta diversity patterns have shown conflicting results. Tessler et al. (2017) observed no correlation between beta diversity patterns generated between metabarcoding and metagenomic analysis of water samples from Brazilian flood plains. In contrast, Latz et al. (2022) found similar beta diversity patterns, despite different taxonomic profiles and richness estimates, between multiple metabarcoding regions and shotgun metagenomics of seawater samples.

In addition to the differences between metabarcoding and metagenomics in contemporary samples, inference of past biodiversity from sedaDNA adds an additional bias—time. After deposition in sediments DNA can be enzymatically digested by microbes or undergo spontaneous degradation and fragmentation, resulting in diminishing quantities of increasingly fragmented DNA surviving through time (Dabney et al. 2013; Orlando et al. 2021). This bias has been shown in a comparison of metabarcoding and metagenomics to reconstruct ancient human oral microbiomes from dental calculus (Ziesemer et al. 2015), where metabarcoding detected species variably over time as a result of length variation among taxonomic groups in the amplified region. Similar patterns have been found when assessing micro‐eukaryotes in ancient marine sediments, with key taxa being missed as a result of length variation in the amplified region (Armbrecht et al. 2021). Furthermore, there was minimal overlap in the detected taxa and limited congruence in the read proportions between methods (Armbrecht et al. 2021). Similar observations were made in Yukon permafrost cores covering the Pleistocene–Holocene transition, with minimal overlap between the taxa detected by metabarcoding and metagenomics (Murchie et al. 2020). Across the few studies comparing metabarcoding and metagenomic of ancient environmental DNA samples we observe variation in the taxa detected (Armbrecht et al. 2021; Murchie et al. 2020; Wang et al. 2021; Ziesemer et al. 2015), but we still lack a full understanding of the detectability of these shared species, and have a limited understanding of any differences in alpha and beta‐diversity metrics over time.

Here we compare metabarcoding and metagenomics of sedaDNA isolated from an archived marine sediment record spanning almost 8000 years collected in the Skagerrak region of the North Sea. We first compared the metazoans detected by both methods, showing differences in detectability across the record. We then analysed the alpha and beta diversity patterns of detected eukaryotic and metazoan taxa, profiling the limitations of each method and providing context for sedaDNA‐derived past biodiversity.

## 2 Methods

### 2.1 Materials and Chronology

**FIGURE 1:**
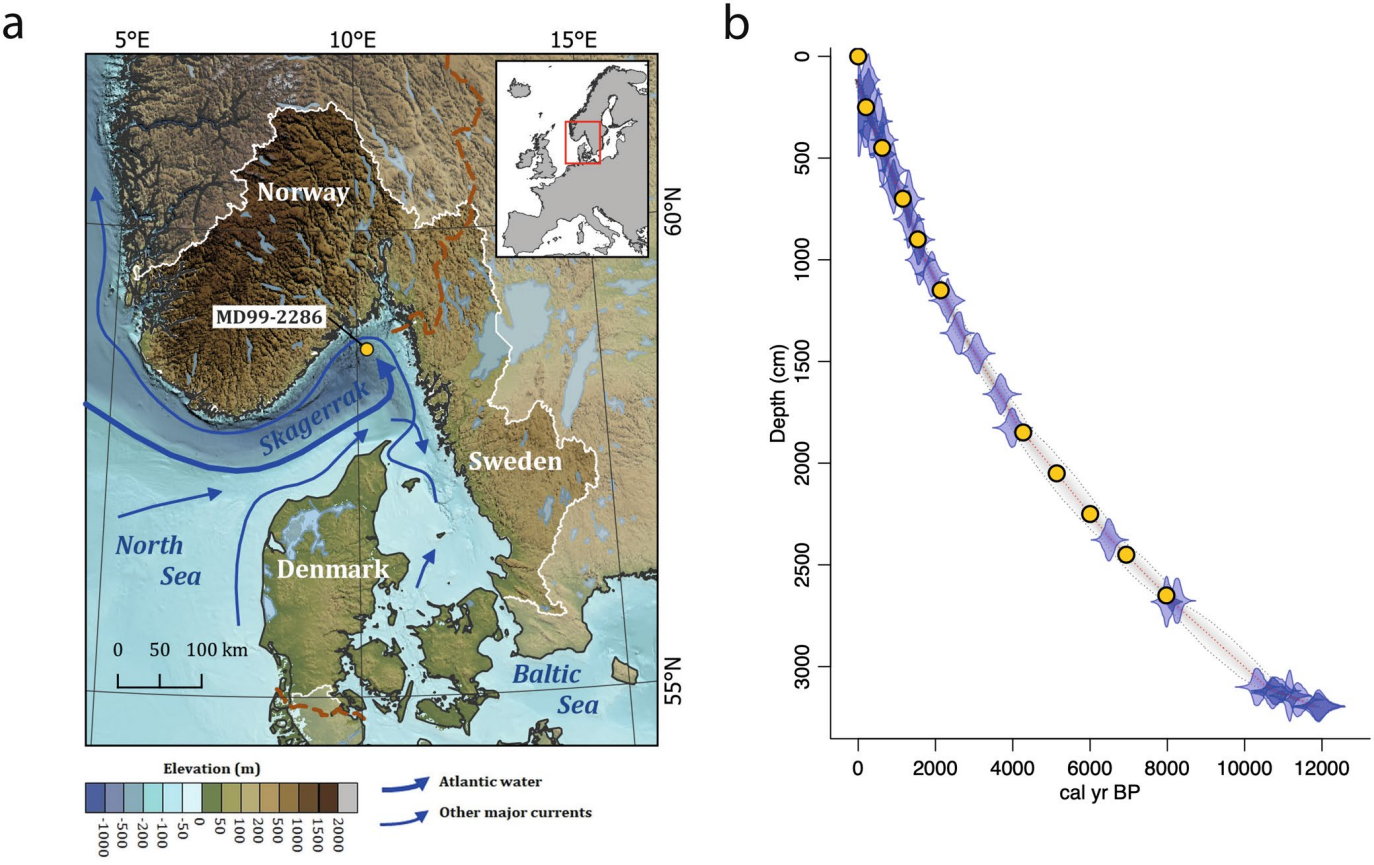
(a) Location of the MD99‐2286 coring site and the modern Skagerrak catchment (white boundary line; dataset ECRINS (2012). Elevation data is from GEBCO Bathymetric Compilation Group (2023), where areas outside the Skagerrak catchment are lightly shaded. Major currents are indicated with arrows (Gyllencreutz et al. 2006). (b) Bayesian age‐depth model outputs for MD99‐2286 core generated using Bacon in R. C14 dates (blue violins) are plotted against core depth (cm) and calibrated years before present (BP), the age‐depth model output is shown as grey shading with the 95% confidence intervals shown with dashed grey lines and the model mean shown with a red dashed line. Samples are shown with yellow points.

The MD99‐2286 sediment core was sampled in 1999 using the giant piston corer *Calypso* onboard the *R/V Marion Dufresne* during the MD114 IMAGES V cruise (details in (Labeyrie and Gherardi 1999)). The recovered core was 32 m in length and was sampled from the southern slope of the Norwegian Trench at 225 m water depth (58.7295, 10.2052, see Figure 1). The split core was stored, sealed in plastic, maintained in a temperature controlled facility at 4°C and was subsampled for eDNA analysis on 25 and 26 October 2021. Subsampling followed established methods (Heintzman et al. 2023) to ensure minimal cross‐contamination of material, collecting sediment from the centre of the core with no material from the uncovered surface of the sediment. Subsamples were stored at −20°C until DNA extraction. A total of 11 subsamples were selected spanning the length of the core. These samples targeted two cultural transitions in the region, the transition to agriculture (6 samples between 4000 and 8500 years before present (BP)) and modern industrialisation (5 samples between present and 2000 years BP). An age‐model for the core was generated using 30 previously published AMS ^14^C dates (Gyllencreutz et al. 2006). A Bayesian model was generated in R (v.4.2.3) (R Core Team 2022) using the package *Bacon* (v3.2.0) (Blaauw and Christen 2011), with the MARINE20 calibration curve (Heaton et al. 2020), no regional offset (∆R) to the MARINE20 curve was applied as there is evidence that ∆R varies across time in the region (Bondevik et al. 2006).

### 2.2 eDNA Extraction

All pre‐PCR laboratory steps were conducted in dedicated ancient DNA facilities, following standard ancient DNA protocols (Heintzman et al. 2023). Extractions were processed using an automated ancient eDNA workflow built on the Qiagen MagAttract Power Soil Pro kit (Qiagen GmbH, Germany), described in (Laine et al. 2024). For each sample 0.4 g of material was transferred to SAFE 2D barcoded 2 mL tubes (LVL technologies GmbH, Germany) containing 200 μL Omni 0.1 mm and 0.5 mm Ceramic Bulk Beads (Omni International, GA, USA) and lysis buffer. All samples were then homogenised on a FastPrep‐96 (MP Biomedicals, CA, USA) with two runs at 1600 rpm for 30 s, interspaced by a 30 s break, samples were then incubated at 37°C overnight with constant homogenization at 1000 rpm. DNA was extracted on a Tecan Fluent DreamPrep 780 (Tecan Trading AG, Switzerland) with the following modifications to the Qiagen MagAttract Power Soil Pro kit recommended protocol (version date 09/2022): QSB1 binding buffer was modified by combining QSB1 buffer, 5 M sodium acetate and 5 M sodium chloride at a ratio of 189:10:1, respectively. To increase the retrieval of smaller DNA fragments the post digestion lysate was combined with modified QSB1 binding buffer and MagAttract Suspension G beads at a 1 to 3 volume ratio, processing 240 μL of lysate for purification. This purification was performed in duplicate to produce two DNA extracts for each sample. The final elution was performed using 60 μL of solution C6. An extraction negative control was included alongside the samples. Metabarcoding and metagenomic library preparation was conducted on each of the duplicate sample DNA extracts and negative extraction control as described below.

### 2.3 Metabarcoding

All laboratory pre‐PCR steps were manually conducted in specialised clean ancient DNA facilities. Two regions were initially targeted for metabarcoding, a variable length (90–130 base‐pairs (bp) without primers) region (V9) of the eukaryotic nuclear 18S ribosomal RNA gene that targets eukaryotes and variably amplifies some bacteria and archaea (Euk1391f, EukBr from (Amaral‐Zettler et al. 2009; Stoeck et al. 2010)) and a ca. 97 bp (without primers) region of the mitochondrial 12S ribosomal RNA that targets vertebrates (Riaz et al. 2011); they are hereafter referred to as 18S and 12S, respectively. For each of the 11 extracted sediment samples and the negative extraction control, eight independent PCR replicates for each primer set were generated. Each of the 20 μL PCR replicate reactions consisted of 2 μL of DNA template, 10 μL AmpliTaq Gold 360 Mastermix (Invitrogen, Waltham, MA USA) and 0.5 μM of each forward and reverse primer with 5′ end unique dual nucleotide tags (8 nucleotides in length, > 3 differences between tags, PCR replicates individually tagged). PCR was conducted with an initial denaturation at 95°C for 10 min followed by PCR cycles of 95°C for 30 s, primer specific annealing (57°C for 18S, 51°C for 12S) for 30 s and 72°C for 60 s. For the 18S and 12S primer sets, we used 26 and 40 PCR cycles, respectively. The appropriate number of PCR cycles was determined by a preliminary qPCR trial using sedaDNA samples (from an unpublished study) known to contain higher DNA concentrations compared to the Skagerrak samples. In this trial 20 μL qPCRs consisted of 2 μL of extracted eDNA, 10 μL AmpliTaq Gold 360 Mastermix, 1 μL of SYBR Green/ROX solution (one part SYBR Green I nucleic acid gel stain (Invitrogen), four parts ROX Reference Dye (Invitrogen) and 2000 parts high‐grade DMSO), and 0.5 μM of each primer. The qPCR was conducted with an initial denaturation at 95°C for 10 min followed by 45 PCR cycles of 95°C for 30 s, primer specific annealing (57°C for 18S, 51°C for 12S) for 30 s and 72°C for 60 s, followed by a dissociation curve. PCR products were visualised on 2.0% agarose gels, quantified according to the brightness of each band, and normalised into three amplicon pools per primer set such that PCR replicates from individual samples were spread across pools. The six amplicon pools were then purified using MagBio HighPrep magnetic beads under the manufacturer‐supplied protocol with 1.8× beads to pool volume for 18S and a final elution of 37.5 μL Qiagen EB buffer. The 12S pool was double size selected using the MagBio beads as this marker produced non‐specific large (500 bp) non‐target products. The double size selection was performed by cleaning the product with beads at 0.8×, retaining the supernatant after placing the mix on a magnetic rack, and the addition of a further 1.0x (to the original product volume) followed by standard cleaning and elution into 37.5 μL Qiagen EB buffer. The six amplicon pools were then quantified and PCR‐free Tagsteady sequencing libraries constructed according to Carøe and Bohmann (2020), ligating Illumina sequencing adaptors which contained 10‐nucleotide dual indexes for each library. The six amplicon libraries, and a library build negative control, were purified using MagBio HighPrep magnetic beads under the manufacturer recommended protocol with 0.9× beads to library volume. All libraries were then quantified using the NEBNext Library Quant qPCR Kit using the manufacturer supplied protocol and the experimental samples were pooled for sequencing at a 2:1 ratio for 18S:12S as the 18S primers amplify much greater taxonomic diversity (Riaz et al. 2011; Stoeck et al. 2010) and thus require more sequencing effort. The final sequencing library pool was sequenced using an Illumina MiSeq instrument with 10% PhiX using a V3 paired 300 bp sequencing kit.

### 2.4 Metabarcoding Bioinformatics

Raw Illumina demultiplexed pools were demultiplexed by primer set and PCR replicate using Cutadapt (v4.2) (Martin 2011). Specifically, PCR replicates were demultiplexed by matching both the forward and reverse tags and the primer sequences for each pair of reads with only a single mismatch permitted across each tag‐primer combination. Since the library preparation method used here produces libraries with amplicons in both orientations, the demultiplexing was run twice, targeting a different primer at the beginning of the forward and reverse reads. Each set of sequences in a different orientation were subsequently processed independently. Both sequenced target fragments were shorter than the total read length so Cutadapt was used to strip primers from the 3′‐end of each read, again with a single error allowed in each read direction. The remaining sequences were then processed using DADA2 (v1.29.0) (Callahan et al. 2016) in R (v.4.2.2) (R Core Team 2022) under default settings unless detailed here. The *filterAndTrim* function was set to filter reads with the following parameters ‘maxN=0, maxEE=c(1,1), truncQ=2’. After ASV generation and chimera removal, the sets of reads from each orientation were combined by generating the reverse complement of ASVs from one orientation, merging the two ASV tables and summing ASVs with 100% identity. Taxonomic assignments of ASVs were generated using blastn (v.2.12.0+) (Camacho et al. 2009) against the NCBI nt database (downloaded 27 Jan 2022) to return 200 hits (−num_alignments 200) per ASV; these were then parsed using a custom R script that uses a lowest common ancestor approach to assign a taxonomy to each ASV (ParseTaxonomy, doi:10.5281/zenodo.4671710). A second set of assignments were generated for the 18S ASVs using the *assignTaxonomy* function from DADA2 with the PR2 database (v.5.0.0) (Guillou et al. 2013). This curated protist‐oriented database, combined with the RDP classifier (Wang et al. 2007), the underlying algorithm of DADA2's *assignTaxonomy*, provides a greater proportion of classifications at higher taxonomic levels, and was used to subset the metabarcoding data to include only *metazoa*. To reduce false‐positive detections, singletons were removed from raw replicate‐by‐sequence tables by filtering any observation (reads per ASV in each replicate) with one read to zero. Then for each ASV, the worst case false‐positive sequence abundance was determined by the maximum number of sequences per ASV across all negatives. As sequences in negative controls are proportionally overrepresented compared to sequences in samples , sequence numbers in negative controls represent a conservative ‘floor’ below which to discard observations. Accordingly, experimental PCR replicates with a lower number of sequences per ASV than the maximum observed number of sequences in the negative controls (extraction, PCR or library negative controls) were set to zero. Next, to exclude non‐target amplification, any ASVs observed to be smaller than 75 bp or larger than 140 bp (12S) or 150 bp (18S) were removed from the dataset. A rarefied dataset was created using the function *rrarefy* from the package vegan (v2.6–4) (Oksanen et al. 2023), selecting 20,000 sequences, and removing replicates with less than this number of sequences. A second normalised dataset was created using cumulative sum scaling implemented in the R package *metagenomeSeq* (v1.40.0) (Paulson et al. 2013).

### 2.5 Metagenomic Library Preparation

Metagenomic double‐stranded library preparation was performed on each of the 11 sample extracts and an extraction negative control. Furthermore, a library build negative control was included. All steps were conducted using the Tecan Fluent DreamPrep 780. Library preparation was carried out with a blunt‐end ligation following Meyer and Kircher (2010). For each reaction, an initial end‐repair reaction was performed using 21.25 μL of extracted eDNA, 2.5 μL of NEBNext End Repair Reaction Buffer and 1.25 μL of NEBNext End Repair Enzyme Mix (New England Biolabs, Ipswich MA, USA), held at 12°C for 20 min followed by 37°C for 15 min. Each reaction was cleaned using the Magbio HighPrep PCR System (MagBio Genomics Inc. Gaithersburg, MD, USA) with the following modifications: before use, beads were washed twice using 20:1 volume of modified Qiagen EB Buffer (1:2 Tween 20:EB buffer) and resuspended using modified EB equal in volume to the uncleaned bead input. The entire end‐repair reaction was combined with 10 μL of washed beads and 500 μL of modified Qiagen PB buffer (pH 5.0, final concentration of added reagents: 0.087 M Sodium Acetate, 0.012 M Sodium Chloride, 1x Phenol Red) and was washed twice using ethanol diluted to 80% concentration with Qiagen PE buffer. The cleaned product was eluted using 18 μL of Qiagen EB Buffer. For each adapter ligation reaction, 17 μL of cleaned end‐repair product, 5 μL of NEBNext Quick Ligation Reaction buffer, 2.5 μL NEB Quick T4 Ligase where combined with 0.5 μL of adapters (25 μM stock), held at 20°C for 30 min. This ligation product was then cleaned using MagBio beads as above with 24 μL final elution. Finally, a fill‐in reaction was performed on the cleaned, ligated product in which a total of 23.5 μL of product was added to 3 μL NEB ThermoPol Reaction Buffer, 1.5 μL *Bst* polymerase (8000 units/mL) and 2 μL of dNTPs (2.5 mM stock), the reaction was held at 65°C for 20 min, 80°C for 20 min and held at 4°C until quantification.

To estimate the number of cycles required in the subsequent indexing PCR, for each library, a qPCR was conducted with 1 μL of fill‐in reaction product, 10 μL Roche LC480 Master Mix (Roche Holding AG, Basel Switzerland) and 0.5 μM each of forward and reverse primer targeting a region ligated in the previous reaction. The qPCR consisted of an initial hold at 95°C for 10 min followed by 30 cycles of 95°C for 30 s, 55°C for 30 s and 72°C for 30 s with fluorescent measurement at each cycle. This was followed by 95°C for 30 s, 55°C for 30 s and a slow ramp from 55°C to 95°C with fluorescent measurement to obtain a dissociation curve. The result of the qPCR showed that 11 cycles was appropriate for the following index‐PCR amplification.

An indexing PCR was conducted with 21 μL of the fill‐in reaction product, 25 μL of NEBNext Q5U MasterMix and primers (0.5 μM each primer in the final reaction) consisting of Illumina sequencing adapters, unique dual indexes (10 nucleotides in length), and a region targeting the ligated region from the earlier ligation reaction. The PCR was held at 98°C for 45 s followed by 11 cycles of 98°C for 15 s, 65°C for 30 s and 72°C for 30 s, a final extension at 72°C was performed for 1 min and the final product held at 4°C until clean up. The PCR product was cleaned using manufacturer standard MagBio HighPrep PCR clean‐up beads under the recommended protocol with a 1:1.6 ratio of PCR product to beads. The cleaned library was eluted in 35 μL Qiagen EB buffer, and then quantified and the library size analysed using the Agilent NGS fragment kit on the 5300 Fragment Analyser according to the manufacturer supplied protocol. Following quantification the resultant libraries were equimolarly pooled and 1 μL of each of the libraries created from extraction and library build negative controls were added to the final pool. The final sequencing library was sequenced on an Illumina NovaSeq 6000 with a single lane of a S4 flowcell (paired 100 bp).

### 2.6 Metagenomic Bioinformatics

Raw Illumina demultiplexed metagenomic read pairs were merged and filtered using fastp (v.0.23.2) (Chen et al. 2018) with the following settings ‘‐V ‐detect_adapter_for_pe ‐D ‐dup_calc_accuracy 6 ‐g ‐x ‐q 30 ‐e 25 ‐l 30 ‐y ‐c’ to remove adaptors and short, erroneous and low complexity sequences. Further uninformative low complexity reads were removed using the preprocess module of sga (v.0.10.15) with a dust threshold set to 3 (Simpson and Durbin 2012). The merged, filtered reads were then mapped using bowtie2 (v2.4.2) (Langmead and Salzberg 2012) under default settings and ‐k set to 5000 to the full NCBI RefSeq (release 213), NCBI nt database (downloaded September 2022) and the Arctic plant and metazoan database from (Wang et al. 2021). The mapped reads were parsed using metaDMG (v.0.38) (Michelsen et al. 2022) and the integrated ngsLCA algorithm (Wang et al. 2022) to taxonomically classify every reads and estimate their post‐mortem DNA damage (damage‐mode: lca, min‐similarity‐score 0.95, max‐similarity‐score 1.00). Raw metagenomic outputs from metaDMG contain read classifications at all taxonomic levels, however we considered only those at the genus level for comparison. Accordingly, the raw data was subset to include only observations at genus with 100 reads or more, in line with evidence that observations approximately below this value carry higher false‐positive rates (Michelsen et al. 2022), this dataset was subset again to include only metazoan genera.

### 2.7 Metagenomic Age‐Damage Model

We developed a model that correlates sediment age with DNA damage. It assumes that once cells or DNA are lost into the environment, DNA repair mechanisms stop and the DNA is subjected to various chemical and physical alterations. Among these changes is depurination, which leads to DNA fragmentation and subsequent deamination of the cytosine bases. This chemical alteration transforms cytosines (C) to uracil, which are subsequently read by the non‐proof reading enzymes in the laboratory as thymine (T), resulting in adenine (A) being incorporated in the complementary base position during PCR amplification. These changes result in a deaminated C being read as a T, and the frequency of this C to T change can then be subsequently measured per taxa in a metagenome as used as a metric of damage (Michelsen et al. 2022; Orlando et al. 2021). The rate of the DNA damage process in eDNA is related to both the pathway of eDNA from source to sink (also known as the ecology of eDNA) (Barnes and Turner 2016) and the diagenetic history of the sedimentary context from which the eDNA is isolated (Dabney et al. 2013). Here, we used the amplitude of DNA damage from metaDMG, restricting to taxa with 500 reads or more and a significance *z* value of 2 or more, and parsing only terrestrial plants (Viridiplantae excluding *Zostera* sp. the only marine plant species detected) at genus level to make a numerical assessment of damage and its variation by age. We used the minimum values from this model to filter the remaining dataset in order to ensure that all taxa were confined to expected conservative minimum damage.

### 2.8 Statistics

Comparisons between ASV and genera richness from metabarcoding and metagenomics were tested using least‐square regression in R. Non‐metric multidimensional scaling ordination was implemented using the *metaMDS* function on Bray‐Curtis and Jaccard dissimilarities from the R package vegan. Procrustes analyses of ordinations were implemented using the *protest* function from the R package vegan. Mantel tests between distance matrices from metabarcoding and metagenomics datasets were conducted using the *mantel* function from the R package vegan with 10,000 permutations for significance testing. All distance matrices were generated using datasets standardised to relative frequencies, bray‐curtis indices were calculated using the number of positive reps for metabarcoding and the relative abundance for metagenomics.

## 3 Results

### 3.1 Sequencing and Taxonomic Assignment

The metabarcoding sequencing produced 21.5 million paired reads with 9,871,769 and 7,171,182 paired reads for the 18S and 12S gene fragments, respectively. There were an average of 84,093 ± 64,149 (SD) paired sequences per PCR replicate for 18S and an average of 51,674 ± 111,364 (SD) sequences per PCR replicate for 12S. Negative controls contained an average of 117 ± 245 (SD) and 11 ± 49 (SD) sequences per PCR replicate for the 18S and 12S datasets respectively. One PCR replicate from a negative extraction control had a large number of sequences (135,227) all assigning to human in the 12S dataset. This PCR replicate was excluded when calculating the reported mean and standard deviation statistics.

After ASV filtering and clustering the 11 samples in the 18S dataset contained 7000 ASVs, of which 714 could be assigned to metazoa using the PR2 database. In contrast, the 12S dataset contained 598 ASVs, of which only nine ASVs had a match with greater than 99% and 95% coverage. From these ASVs only one had a non‐human metazoan match: a total of 21,169 sequences found in a single PCR replicate (from 5637 cal. year BP) corresponding to the Family *Clupeidae* (herrings and sprats). We interpret this lack of data as a failure to reliably amplify the target gene fragment for vertebrates. Electrophoresis gel imaging of PCR products showed limited amplification and non‐specific amplification products, therefore, all subsequent analyses were conducted using the 18S dataset. Negative controls from the 18S dataset contained 23 ASVs with high‐quality matches (> 99% identity, > 95% coverage) to reference databases (NCBI nt). These ASVs had a total of 1975 sequences across all negative controls, and none could be assigned a taxonomic identity below family.

**FIGURE 2:**
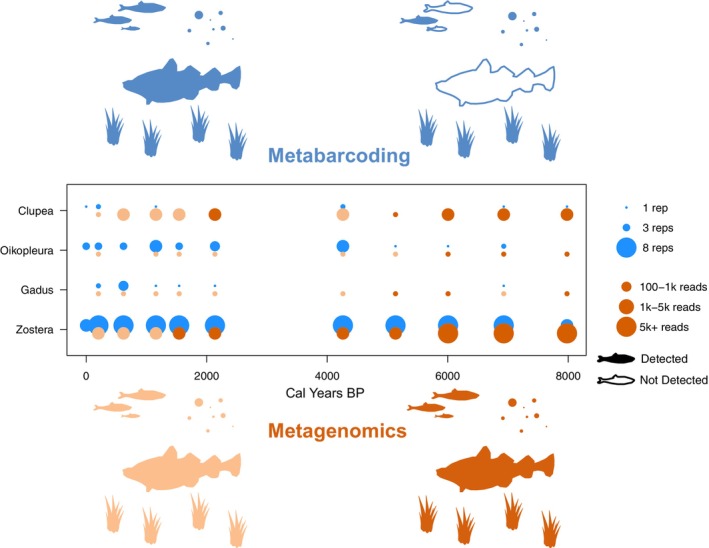
Detections of overlapping metazoa (Clupea ‐ herring, Oikopleura ‐ larvacean, Gadus ‐ cod) and eelgrass (Zostera) from metabarcoding and metagenomic sequencing of 11 marine sedaDNA samples from the Skagerrak (North Sea). The central box shows detections using both methods, the size of the bubbles indicates the number of positive PCR replicates (metabarcoding) and the number of mapped reads (metagenomics). The bubbles in dark orange indicate the detections that had sufficient post‐mortem damage to be characterised as damaged according to the age‐damage model described in the main text, the light orange bubbles indicate those which do not exhibit damage patterns. The coloured fish and eelgrass icons indicate a simplified overview of the detected community from recent (< 2000 years) and early Holocene material, with the metabarcoding shown in blue and the metagenomics shown in orange.

The metagenomic sequencing produced 4.98 billion reads, with an average of 226,384,537 ± 31,200,373 (SD) paired reads per experimental sample and an average of 25,236 ± 12,412 (SD) for negative control samples. A total of 267 genera were detected across the 11 sediment samples before filtering for damaged reads. Negative control samples had more than 100 sequences assigned per sample to only two soil bacteria genera (261 reads to *Mesorhizobium*, 499 reads to *Bradyrhizobium*). Post‐filtering, 87 observations from 17 taxa remained, including two non‐aquatic taxa (*Homo, Loxodonta*), which were excluded from downstream analysis (see Supporting Information 1; Figure S1.1–S1.3). Three metazoan genera overlapped between the metabarcoding and metagenomic datasets (*Oikopleura*—larvacean, *Gadus*—cod, *Clupea*—herring), with variable but sufficient resolution for genus level assignments of metabarcoding ASVs as discussed in Supporting Information 2. These detections are shown in Figure 2 above; while not a metazoan genera, we include *Zostera* in these comparisons as it is a genus of eelgrass with a key structural and functional role in marine ecosystems (Boström et al. 2014), and it appears in both the metabarcoding and metagenomic datasets with persistent detection across the period. The age‐damage model authenticated 17 observations across seven metazoan genera as having sufficient evidence of DNA damage to be considered ‘ancient’; see Supporting Information 3 and 4 for age‐depth (Figure S3.1) and age‐damage models (Figure S4.1). Among the three metazoans detected in both datasets, observations could be authenticated as damaged from 6000 cal. year BP back through time (see Supporting Information 5, Figure S5.1 for example damage plots). The *Zostera* detections had sufficient evidence to be designated as damaged under the age‐damage model earlier than 2000 cal. year BP.

### 3.2 Diversity Comparisons

**FIGURE 3:**
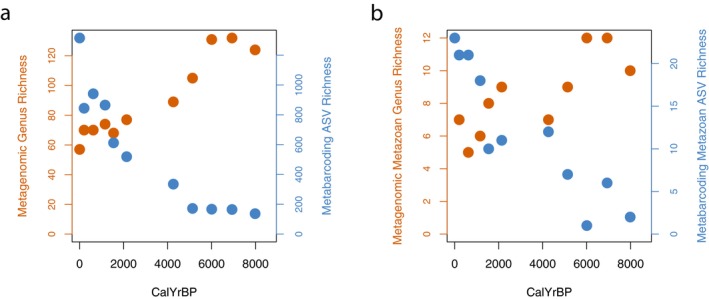
Total number of detected metagenomic genera (red) and metabarcoding ASVs (blue) from marine sediments for (a) all non‐viridiplantae detections and (b) only metazoan genera against calibrated age in years before present.

The metagenomic data showed decreasing genus richness towards the present when considering both all taxa and only metazoa (Figure 3). Conversely, the metabarcoding data showed the opposite pattern, with ASV richness decreasing back through time for both the entire dataset and the data subset for metazoa (Figure 3). Furthermore, this pattern was consistent regardless of normalisation technique employed or data filtering stringency (see Supporting Information 6; Figure S6.1–S6.3). Comparisons between all four datasets shown in Figure 3 revealed significant (*p* > 0.05, see Supporting Information 7; Figure S7.1), strong, negative relationships between ASV and genus richness measured by the two techniques.

**FIGURE 4:**
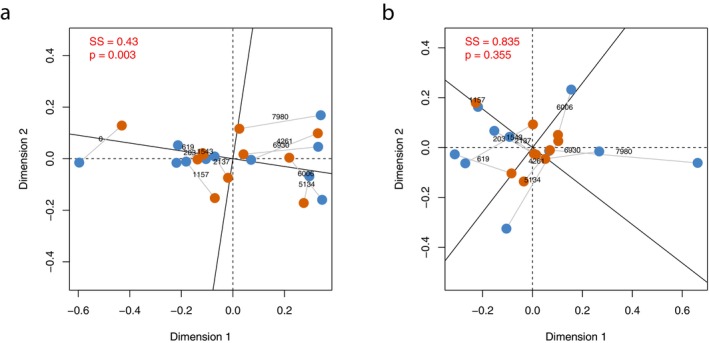
Non‐metric multidimensional scaling plots generated using Bray‐Curtis dissimilarities for (a) all non‐viridiplantae data and (b) metazoan data. Metabarcoding is shown in blue and metagenomics is shown in orange. The ordinations have been superimposed using a procrustes analysis with the original metagenomic axes shown with solid black lines and the metabarcoding axes shown with dashed lines. Solid grey lines connect the points for each sample and the age of the sample in calibrated years BP is shown at the centre of each connecting line. The sum of squares and *p* value statistics for each procrustes test are displayed in red at the top left of each plot.

Ordinations of samples showed continuous separation through time for datasets containing all ASVs/genera (Figure 4a). This pattern was broadly similar across both metric and non‐metric multidimensional scaling ordinations generated using both Jaccard and Bray‐Curtis similarities (Supporting Information 8; Figure S8.1–S8.4). In contrast, patterns were less clear across datasets subset for metazoans (Figure 4b), with some ordinations showing similar separation through time to all ASV/genera datasets (see nMDS Bray‐Curtis metabarcoding in Supporting Information 8; Figure S8.1), and others showing no clear pattern separating samples by time (see MDS Bray‐Curtis metagenomics in Supporting Information 8; Figure S8.3). In line with these findings, across all ordinations tested there was a significant (procrustes analysis, *p* < 0.01) correlation between metabarcoding and metagenomic datasets including all ASVs/genera. In contrast, when considering any comparison with a dataset subset for metazoans there was no consistently significant correlation (procrustes analysis, *p* < 0.05) between metabarcoding and metagenomic datasets across all ordinations, with significance found in some comparisons and not in others (Supporting Information 8—Data S1). Mantel tests conducted between metabarcoding and metagenomic datasets showed similar patterns to the procrustes analysis, with significant correlations (*p* < 0.01) only between the non‐subset datasets (full model outputs for all comparisons in Supporting Information 9; Table S9.1) (Figure 4).

## 4 Discussion

Here we compared metazoan biodiversity generated from metabarcoding and metagenomic analyses of an archived marine sediment record. We show that for overlapping metazoan taxa and eelgrass (*Zostera*), detection is broadly similar between metabarcoding and metagenomics across the most recent ca. 2000 years. In the older portion of the record, metagenomics detects the overlapping taxa more reliably, with many metabarcoding false‐negative detections. We then demonstrate divergent patterns of ASV and genus richness between the technologies with metabarcoding showing a decrease in ASV richness back through time, compared to an increase in detected genera using metagenomics. Finally, we highlight that beta dissimilarity of samples across the core is roughly similar when considering all ASVs/genera, but that divergent results were shown with metazoan subsets of the data, with limited congruence considering only metazoa. Overall, our results underline the importance of understanding the differences between detections from metabarcoding or metagenomics before making ecological inferences to understanding biodiversity change from sedaDNA records.

Our data comparing the taxa detected by metabarcoding and metagenomics prompt two key questions: firstly, why do we detect different taxa? And secondly, why do we detect some taxa differently?

The first question is easily approached, as a series of well documented biases affect all DNA datasets. The most obvious is metabarcoding primers; different primers detect different species as mismatches to the target sequence can produce biases in amplification (Deiner et al. 2017; Elbrecht et al. 2019; Taberlet et al. 2018). In contrast, metagenomics can theoretically detect any taxon in the total DNA pool given sufficient sequencing depth, but even substantial sequencing effort can miss taxa with low concentrations of environmental DNA (Stat et al. 2017; Zimmermann et al. 2023). Another key reason for differences between metabarcoding and metagenomics results is the incompleteness of DNA reference databases. For metabarcoding, DNA reference barcode coverage of European taxa for the best sequenced marker region (a region of cytochrome c oxidase subunit I) is below 50% for almost all taxonomic groups (Weigand et al. 2019). For taxonomic assignment of our metabarcoding data, we used both the highly curated PR2 protist database as well as the large and relatively uncurated NCBI nt database. Thus metabarcoding ASVs that we detected but that do not have a reference barcode were likely to be assigned to a close relative or higher taxonomic level. Metabarcoding targets single conserved taxonomically informative gene regions, but many genomic regions also have sufficient resolution to provide accurate taxonomic assignments (Breitwieser et al. 2019; Huson et al. 2016). Metagenomics leverages these taxonomically informative regions but as evidenced by the majority of unassigned reads, much of the sequencing effort remains unassignable even when genomic references are available. Despite recent sequencing efforts (Lewin et al. 2018), we lack high‐quality reference genomes for the vast majority of the tree of life, and thus, we remain unable to detect large proportions of total diversity and can only produce a low‐resolution taxonomic overview of ecosystems using metagenomes. Collectively these biases mean that metabarcoding and metagenomic approaches detect different subsets of total biodiversity, with incomplete overlap of the taxa detected (Armbrecht et al. 2021; Murchie et al. 2020; Tessler et al. 2017; Wang et al. 2021). We show similar results in our data, with very different profiles of taxonomy detected in terms of identity and richness between metabarcoding and metagenomics. Crucially, we see only three metazoan taxa overlapping between the two methods, despite employing deep metagenomic sequencing and a large number of PCR replicates in metabarcoding.

The second of our key questions, “why do we detect some taxa differently?”, is critical as the false‐negative detections of metazoa shown by metabarcoding of older sediments could be interpreted as a true lack of some taxa in older periods. Ancient DNA is frequently fragmented and has DNA damage that increases through time (Armbrecht et al. 2022; Kjær et al. 2022; Orlando et al. 2021). This is problematic for metabarcoding as PCR amplification, and thus detection, relies on the complete target fragment being present in the sample. Given that the majority of regions targeted by metabarcoding primers in marine sedimentary studies are greater than 150 bp (Nguyen et al. 2023), ancient sedimentary DNA in many cases may simply not contain enough full length fragments for complete amplification of the community. Studies have shown that taxon specific length variation in sequenced amplicons can bias metabarcoding of ancient DNA (Armbrecht et al. 2021; Ziesemer et al. 2015). Here we provide evidence that there is variation in detectability over time, but do not find evidence that length variation has a strong effect on diversity metrics (see Supporting Information 10, Figure S10.1–S10.4). In contrast, metagenomics can provide taxonomic inference from shorter fragments, in some cases only 40‐60 bp (Kjær et al. 2022). Accordingly, remarkably old DNA, of over one million years, has to date only been recovered using metagenomics (Dalén et al. 2023). In our dataset we observe a decrease in the average mapped fragment length of metagenomic reads (Supporting Information 10, Figure S10.2) back through time, but show no associated loss of taxonomic detection (Supporting Information 10, Figure S10.4), providing further evidence that short metagenomic reads can provide accurate taxonomic inference far back in time. We also present metabarcoding of ~150 bp amplicons from sediments dated to almost 8000 years BP, despite the average fragment length of our metagenomic sequences being much less than 100 bp in length (Supporting Information 10; Figure S10.1–S10.2). This evidence, the similarly of our metabarcoding replicates across time (see Supporting Information 11, Figure S11.1), and the routine amplification of long (> 100 bp) amplicon metabarcoding in sedaDNA studies (Nguyen et al. 2023; Romahn et al. 2024), indicates that a small proportion of DNA remains amplifiable through metabarcoding, even in samples many thousands of years old. However, at least in cores of similar age and geochemistry as presented here, beyond 2000 years BP detections of marine metazoa become patchy, while detections from metagenomics remain comparably more consistent.

One of the most conceptually simple metrics of biodiversity is the number of species observed. Our data revealed that two commonly used DNA‐methods for understanding past biodiversity show entirely contrasting patterns of taxonomic richness across the study period. Published alpha diversity comparisons between metabarcoding and metagenomics in modern samples have revealed broadly coherent results between data types (Bista et al. 2018; Poretsky et al. 2014; Tedersoo et al. 2015). Furthermore, an increasing number of studies employ either metabarcoding or metagenomics to document the richness of taxa across time in sedaDNA records (Wang et al. 2021; Zimmermann et al. 2021). Therefore, the discordant species richness patterns between techniques seen here underline the importance of considering the effect of methodological biases that affect biodiversity metrics in ancient DNA. The false‐negative metabarcoding detections (Figure 2) explain at least part of the observed richness patterns, as fewer species are detected in the older sections of the record due to DNA degrading and becoming undetectable. This mirrors findings with other metabarcoding markers when amplifying marine sediments showing a decreasing (but non‐significant) trend in ASV richness back through time (Romahn et al. 2024). The dramatic increase in richness over time observed here by metagenomics occurs at the same period (3000–4000 k years BP) as the loss of richness in metabarcoding. It is uncertain if this richness increase reflects an actual increase in biodiversity. However, the scale (almost a doubling across the record), and co‐occurrence of the increase with a decrease in metabarcoding, suggest a methodological explanation. In order to compare the two methods, the same initial eDNA extract was used for both metabarcoding and metagenomics, however the DNA extraction method used was optimised for ancient DNA (Laine et al. 2024), and thus may enrich smaller fragments. It is conceivable that this bias towards shorter fragments reduces the detectability of longer DNA fragments, which are more common in the more modern sections of the core, when using metagenomics. Indeed, the core top sample showed very low richness, and no detections of the shared metazoa (Figure 2), despite detections in the metabarcoding data. At worst these results indicate that, at least for samples with DNA damage in the range seen here, it is difficult to produce accurate measures of alpha diversity using either metabarcoding or metagenomics. However, based on the true‐positives in the younger section of the core for metabarcoding we suggest that richness patterns for metabarcoding experiments may be informative in marine material which contains DNA with limited damage, here < 2000 years BP. The reverse may be true for metagenomics, as detections in the younger sections of the core may be biased by methodological choices (DNA extraction techniques, sequencing library preparation, bioinformatic methods) that aim to increase the amount of ancient DNA. Overall, we urge caution when interpreting alpha diversity patterns generated from metagenomic analyses of marine sediment cores covering recent periods.

Beta diversity patterns across the core showed a different picture to the alpha diversity patterns, with congruence between the methods in datasets with all taxa. It is unsurprising to see contrasting patterns in metazoan data subsets, as selecting subsets of taxa from any biodiversity dataset would produce a biased picture of beta diversity, which we might expect to differ from the complete picture. While there are some cases of convergent beta diversity across disparate taxonomic groups (Holman et al. 2021), marine communities are strongly structured by size class (Richter et al. 2022), so we should not expect beta diversity patterns of disparate groups (e.g., prokaryotes, metazoa, protozoa) to be similar. Taphonomic and methodological biases also affect beta diversity patterns, although we see similar patterns between methods here. Therefore, when a sedaDNA time series has highly variable damage within the dataset caution should therefore be used before applying ecological explanations for changes in beta diversity.

The patterns of species detection and biodiversity here have been generated from material that has been stored for over 20 years. They thus represent a worst‐case scenario for the detectability of taxa, where freshly collected material would produce the most reliable detections. Previous work has identified that older sediment material does frequently contain contaminants (post‐sampling fungal growth) (Armbrecht et al. 2021; Selway et al. 2022), but that a strong marine signal dominates the recovered biodiversity patterns. We also observe the presence of post‐sampling fungal growth in our record (see Supporting Information 12, Figure S12.1–S12.2), but in congruence with previous work find that the overwhelming signal is that of the environment from which the core was sampled. In line with others (Armbrecht et al. 2021; Selway et al. 2022), we advocate that using the characteristic ancient signal, and subsetting by marine taxa as appropriate, will help produce an accurate reconstruction of marine paleo‐archives.

An outstanding question for researchers using the ancient DNA damage from metagenomic data to subset observations is how much damage is sufficient to classify an observation as ancient? In our study, we assumed that the damage we observed in plant taxa, which have strong cell walls and must have been transported through the water column, represent a good minimum measure against which to compare damage from in situ organisms. However, filtering purely by this metric would omit taxa, particularly in the more recent part of the record, that are likely true‐positive detections (Figure 2—light orange observations). Furthermore, our age‐damage model (Supporting Information 4) does not show a smooth consistent profile, likely as a result of a small number of detected plant taxa. This problem may be further exacerbated in offshore regions where plant material is unlikely to be deposited, or in high latitude ecosystems with limited vegetation. Metagenomics is an emerging and powerful technique to understand changing marine communities (Armbrecht et al. 2022; Zimmermann et al. 2023), and work is needed to help establish models for DNA damage through time in marine sedimentary contexts to enable ancient‐filtering of these complex datasets.

Overall, our study raises critical questions about detection and diversity patterns generated from sedaDNA records, particularly for metazoan species. Over time eDNA will degrade and become undetectable, but as metabarcoding and metagenomics produce different pictures of biodiversity from the same sample, different interpretations are possible using different techniques.

## Author Contributions

L.E.H., M.W.P. and K.B. conceived the ideas and designed the methodology; L.E.H. and G.Z. collected the data; L.E.H. and G.Z. analysed the data; L.E.H. led the initial writing of the manuscript. All authors contributed critically to subsequent drafts and gave final approval for publication.

## Conflicts of Interest

The authors declare no conflicts of interest.

## Supporting information


Data S1.

## Data Availability

All metagenomic and metabarcoding sequencing data are available under ENA accession number PRJEB75437. Intermediate files, metadata, scripts and figures are permanently archived via Zenodo with the DOI doi.org/10.5281/zenodo.11108965. Amplicon sequence variant occurences have been deposited in the Global Biodiversity Information Facility and are accessible at doi.org/10.15468/2ve69k.
