# Supplementary material for: Navigating Past Oceans: Comparing Metabarcoding and Metagenomics of Marine Ancient Sediment Environmental DNA
Source: Mol Ecol Resour. 2025 Feb 20;25(6):e14086. doi: 10.1111/1755-0998.14086 (PMC12225701; doi:10.1111/1755-0998.14086)
Supplement: Supplementary file 1 — Data S1. [file MEN-25-e14086-s001.pdf]

**Supporting Information for:**

# Navigating past oceans: comparing metabarcoding and metagenomics of marine ancient sediment environmental DNA

Luke E. Holman[1] * (0000-0002-8139-3760)

Giulia Zampirolo[1] (0009-0001-0145-1538)

Richard Gyllencreutz[2, 3] (0000-0003-3193-8598)

James Scourse[4] (0000-0003-2658-8730)

Tobias Frøslev [5,6] (0000-0002-3530-013X)

Christian Carøe [7] (0000-0001-9601-6768)

Shyam Gopalakrishnan[8] (0000-0002-2004-6810)

Mikkel Winther Pedersen[6] (0000-0002-7291-8887)

Kristine Bohmann[1] (0000-0001-7907-064X)

1 Section for Molecular Ecology and Evolution, Globe Institute, University of Copenhagen, Denmark

2 Department of Geological Sciences, Stockholm University, Sweden

3 Bolin Centre for Climate Research, Stockholm University, Sweden

4 Centre for Geography and Environmental Science, University Of Exeter, United Kingdom

5 Centre for Ancient Environmental Genomics, Globe Institute, University of Copenhagen, Denmark

6 Global Biodiversity Information Facility, Copenhagen, Denmark

7 Novonesis A/S, Biologiens Vej 2, Lyngby, Denmark

8 Centre for Hologenomics, Globe Institute, University of Copenhagen, Denmark

**Supporting Information 1 - Non-aquatic taxa**

Contamination of modern human DNA into ancient samples is a commonly documented phenomenon. It can represent a real signal in the environment collected contemporaneously with ancient samples or be introduced in the laboratory during extraction, sequencing library preparation or even during sequencing, despite stringent steps being taken to prevent it (Llamas et al., 2017; Whitmore et al., 2023). We therefore chose not to include any ASVs or metagenomic sequences assigned to the genus *Homo*, filtering them out as described in the main methods.

We additionally filtered out metagenomic reads assigned to genus *Loxodonta* (african elephants), as we consider these reads anonymous and uninformative for the experiment conducted here. We detail our investigations into these reads below and acknowledge that there is uncertainty regarding their origin. The findings presented in the main manuscript are in no way affected by the removal of these reads, and we provide the below explanation with a view to helping others whose data show unexpected detections.

*Loxodonta read investigation*

After running metaDMG for taxonomic profiling and post-mortem DNA damage estimation, a total of 431 reads across 10 samples (all experimental, no controls) were assigned to the genus *Loxodonta*. Minimum of 1 read, maximum of 282 (for the oldest layer - MD9-2650). We applied a filtering threshold allowing for a minimum of 100 reads and a MAP_significance above 2, as recommended in Michelsen et al 2022. All the reads assigned to the genus *Loxodonta* were filtered out, except for MD9-2650 which clearly showed damage patterns (MAP_damage = 24 %) and a MAP_significance > 4 (Fig. S1.1).

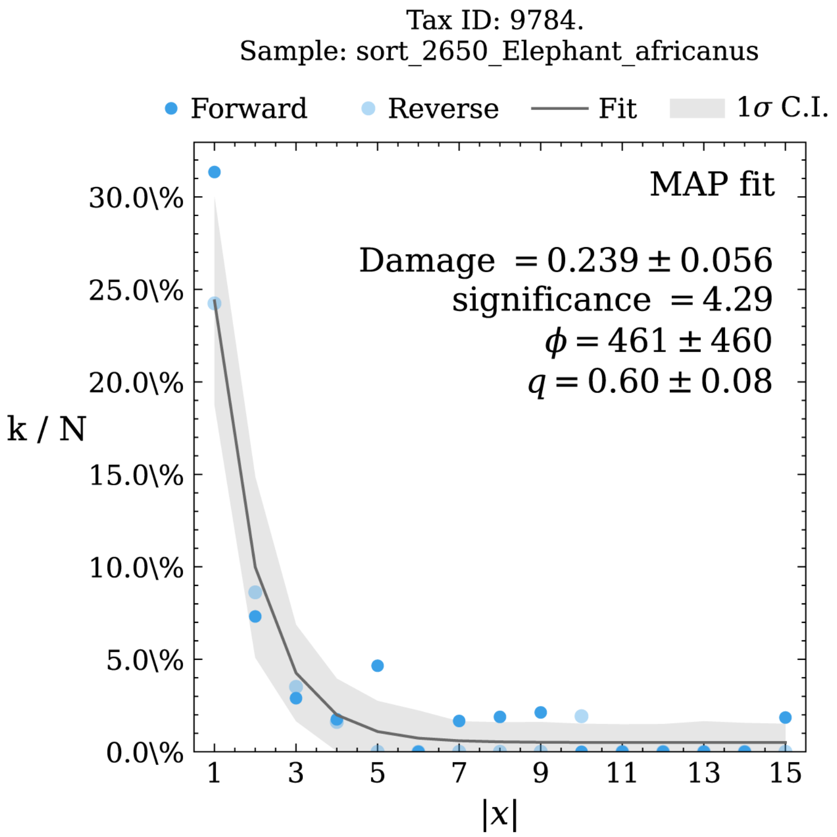


*Figure S1.1: Position (x) of the DNA damage specific nucleotide mis-incorporations (k/N) due to DNA damage onthe forward and reverse strand for reads classified at the genus Loxodonta (sample MD9-2650). Dark-blue dots are C > T transitions, light-blue dots are G > A transitions, gray areas indicate the fit. MAPfit: 0.239 ± 0.056, significance: 4.29, number of reads: 282, meanL: 59.19 ± 19.87.*

The presence of elephant DNA in a sample from a marine core can most likely be interpreted as a false positive assignment. Members of the genus *Loxodonta* are exclusively found in sub-saharan Africa and the signal is therefore 1) a misclassification of reads from a closely related species, 2) a misclassification of reads from an unrelated taxa with a contaminated reference genome or 3) the contamination of samples with a true Loxodonta signal at some point after sample collection and before library sequencing.

To explore the first and second of these possible explanations, we further validated the alignment for sample MD9-2650 (Key et al., 2017), by assessing the coverage distribution, and the edit distance. To eliminate the low likelihood possibility of the *Loxodonta* reads originating from a Mammoth presence in the ecosystem (see (Ukkonen et al., 2011) for details on Mammoth distributions in this area and period) we

proceeded to a further step of authentication of the classified reads. We extracted all *Loxodontae* reads (285) from the lca files produced by metaDMG following (Kjær et al., 2022) and mapped them against the genome assembly of *L. africana* (*Loxafr3.0, Genbank Assembly ID: GCA_000001905.1*) and of *Mammuthus primigenius (MamPri_Loxafr3.0_assisted_HiC*) (Sandoval-Velasco et al., 2023). We first assessed the breadth and depth of coverage for the mapping reads with BAMCOV (v.0.1) and visualised coverage histograms (Fig. S2.2, Supplementary Datasets 3-4). Overall, the alignment to *Mammuthus primigenius* reports a greater number of reads and covered bases distributed across the scaffolds, while the alignment to *Loxodonta africana* shows a lower number of reads mapping to the genome and a lower number of covered bases retrieved for each scaffold (Fig. S1.2). In addition, the latter also presents a greater amount of reads mapping only once to scaffolds (*52* against the 20 from the *Mammuthus primigenius)* (see rawdata/supplementary/ in provided repository). Finally, we assessed the edit distance to the reference based on the assumption that a lower number of mismatches indicates a closer match between the read and the reference, implying a more accurate alignment (Fig. S1.3). The edit distance between reads and reference ('NM' tag from the bam files) for unique alignments was extracted and then the proportion of reads carrying mismatches was plotted (Jensen et al., 2019). Unfortunately, there was no mapping to the mitochondrial genome of either mammoth or elephant, preventing us from additional phylogenetic investigations. The codes and R scripts used are available at doi.org/10.5281/zenodo.11108965.

Overall these investigations show limited evidence that the observed signal originates from a closely related species. Additionally, as reads mapped across many different regions of the genome (Fig. S1.2) we can exclude the possibility of a very small number of contamination contigs or a pile-up of reads in a single location from an erroneously incorporated common gene region. We could not identify any change in reagents or methods in this investigation in relation to successful experiments in the same facility showing no evidence of contaming reads (Laine et al., 2024). We conclude that the source of the contamination in this experiment remains unknown, but reiterate that the results presented in the main manuscript are in no way affected by the omission of the contaminating reads.

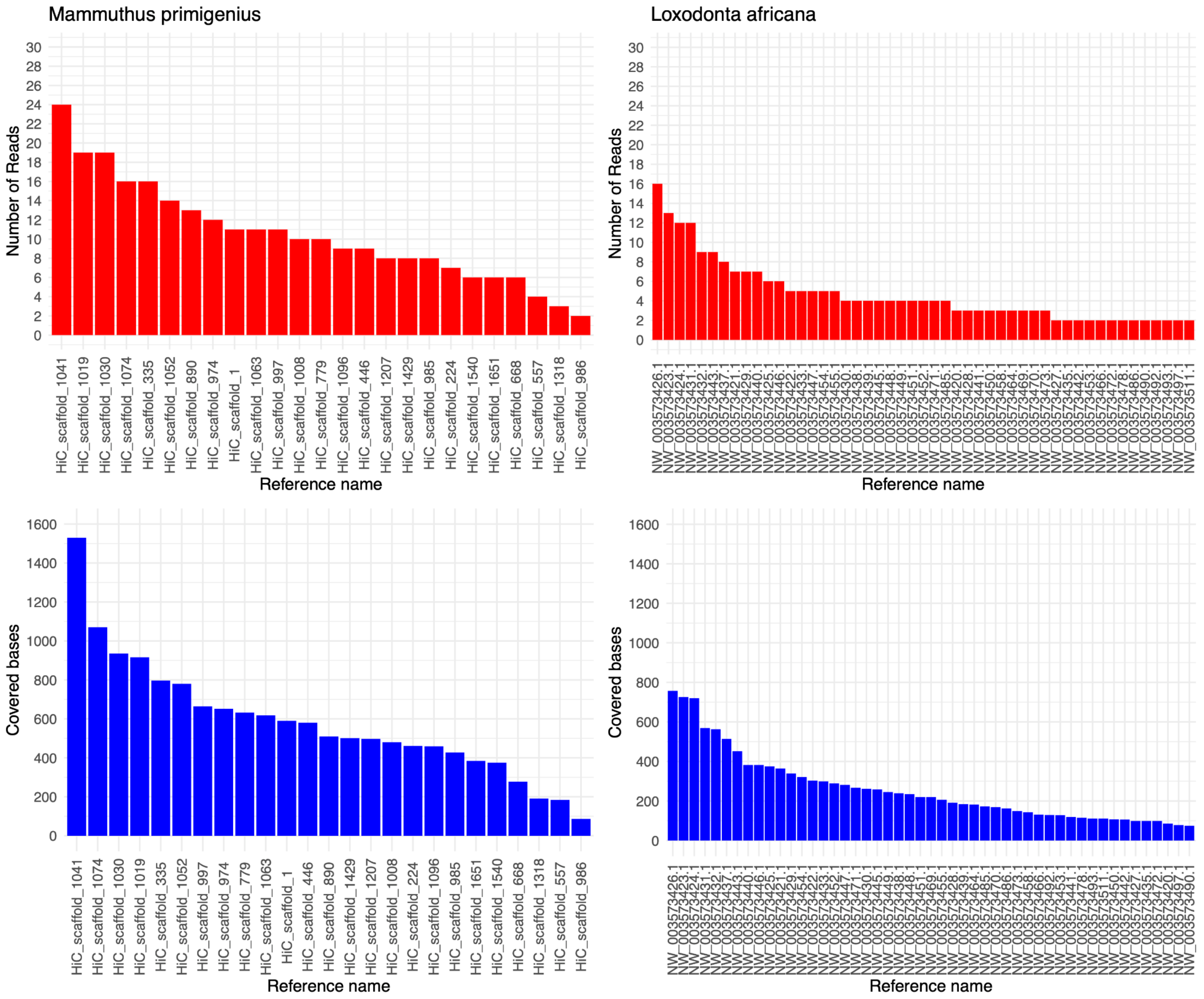


*Figure S1.2 Number of reads (red) and bases covered (blue) from metagenomic sample MD9-2650 mapping to Mammuthus primigenius (left) and Loxodonta africana (right). The figure shows the mapping statistics for a number of reads > 2, 1 read-mappings are not shown in this figure.*

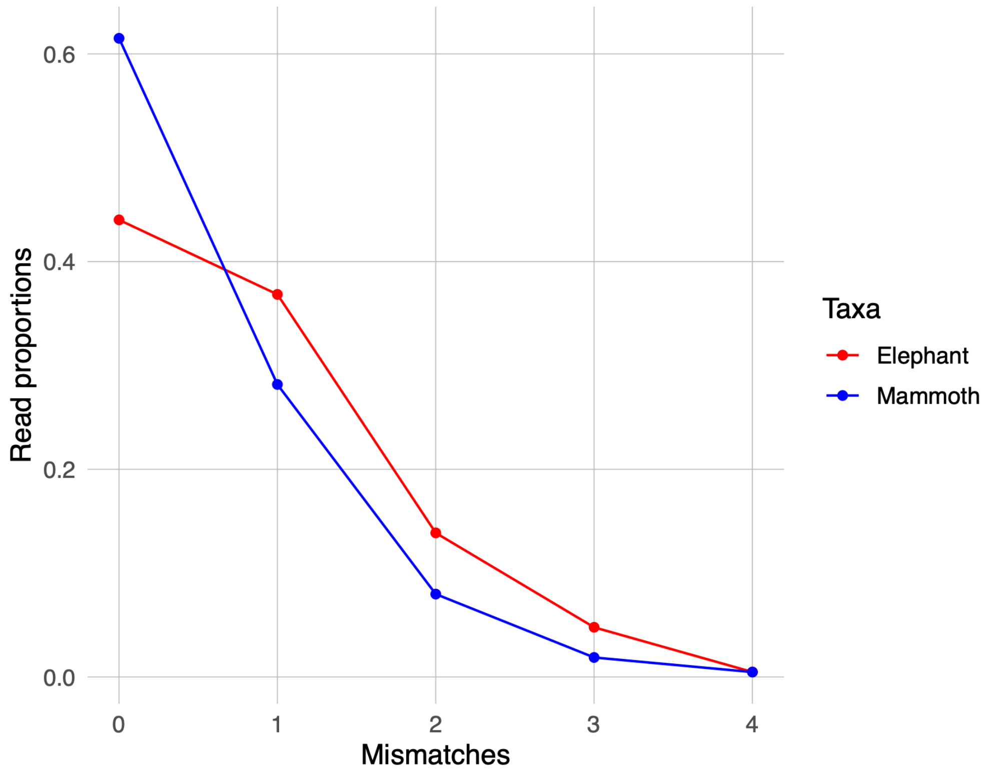


*Figure S1.3: Edit distance for reads mapped from metagenomic sequencing of sample MD9-2650. X-axis shows the number of mismatches, y-axis shows the proportion of reads assigned to the Elephant (red line) and the Mammoth genome (blue line). The reads mapped to the mammoth genome exceed those mapped to the elephant.*

**Supporting Information 2 - Overlapping genus metabarcoding ASV assignments**

There is good evidence that common 18S metabarcoding primers amplify fragments that can only assign species to a minority of observed sequences in marine ecosystems (Latz et al., 2022). The following ASVs were identified as matches for metazoan *genera* in the metagenomic dataset. After being identified as possible matches using the assignment methods outlined in the main manuscript, and after being screened for ASVs with the same taxonomic match, each ASV was again matched against the NCBI nt database using the online NCBI blast portal under default settings on 19th Jan 2024. All matching species were taxonomically confirmed and their habitat and range checked via FishBase (Froese & Pauly, 2024)The justification for each assignment is detailed below.

*ASV_621 Oikopleura*

The ASV has >10 100% identity, 100% coverage hits to sequences produced by multiple research groups for the species *Oikopleura dioica.* The nearest member of the genus (*Oikopleura labradoriensis*) has 91.67% sequence identity. We can confidently assign this ASV as the only member of Oikopleura in the metabarcoding dataset, and the ASV likely corresponds with the species *Oikopleura dioica*.

*ASV_1456* Clupea

The ASV had 100% identity, 100% coverage hits for 12 species. From these, five were freshwater species (*Acrossocheilus fasciatus, Barbus barbus, Onychostoma alticorpus, Onychostoma barbatulum, Onychostoma macroplepis*) and six were marine species not documented in the study area (*Anchoa cayorum, Chanos chanos, Sardinella marquesensis, Talismani antillarum, Xenodermichthys copei*) leaving only *Clupea harengus*. We can confidently assign this ASV as the only member of Clupea in the metabarcoding dataset.

*ASV_2468* Gadus

The ASV had 100% identity, 100% coverage hits for 12 species. From these one was a freshwater species (*Lota lota*), six were marine specie snot documented in the study area (*Antimora microlepis, Coryphaenoides rudis, Gadus chalcogrammus, Gadus macrocephalu, Laemonema barbatulum, Urophycis chuss*) leaving five possible species (*Brosme brosme, Melanogrammus aeglefinus, Merlangius merlangus, Molva molva, Pollachius pollachius*). Within these species three belong to the family *Gadidae* and all belong to the order *Gadiformes*. Notably sequences belonging to the only member of the genus *Gadus* in the study region (*Gadus morhua*) had a single base mismatch to the ASV in all cases. We are confident this ASV represents a cod or cod-like species, we label the ASV as *Gadus* in the main manuscript but acknowledge the uncertainty.

## Supporting Information 3 - Age-depth model

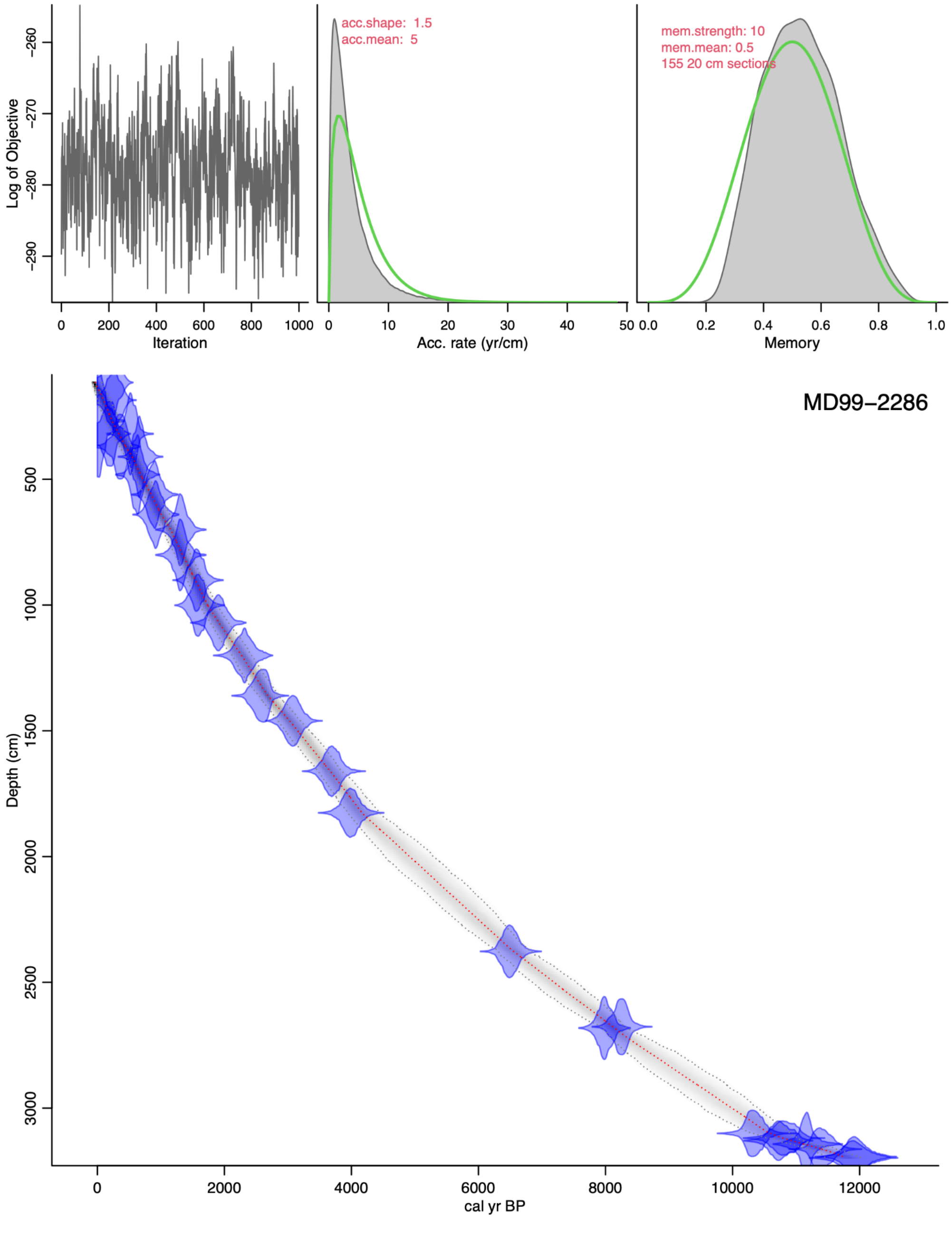


*Figure S3.1: Bayesian age-depth model outputs for MD99-2286 core generated using Bacon in R. Top panel shows MCMC interactions (left), prior(green) and posterior (grey) curves for accumulation rate (centre) and dependence between adjacent accumulation rates in the model (left). C14 dates (blue violins are plotted against core depth (cm) and calibrated years before present (BP), the age-depth model output is shown as a cloud grey shading with the 95% confidence intervals shown with dashed grey lines and the model mean shown with a red dashed line.*

**Supporting Information 4 - Age-damage model**

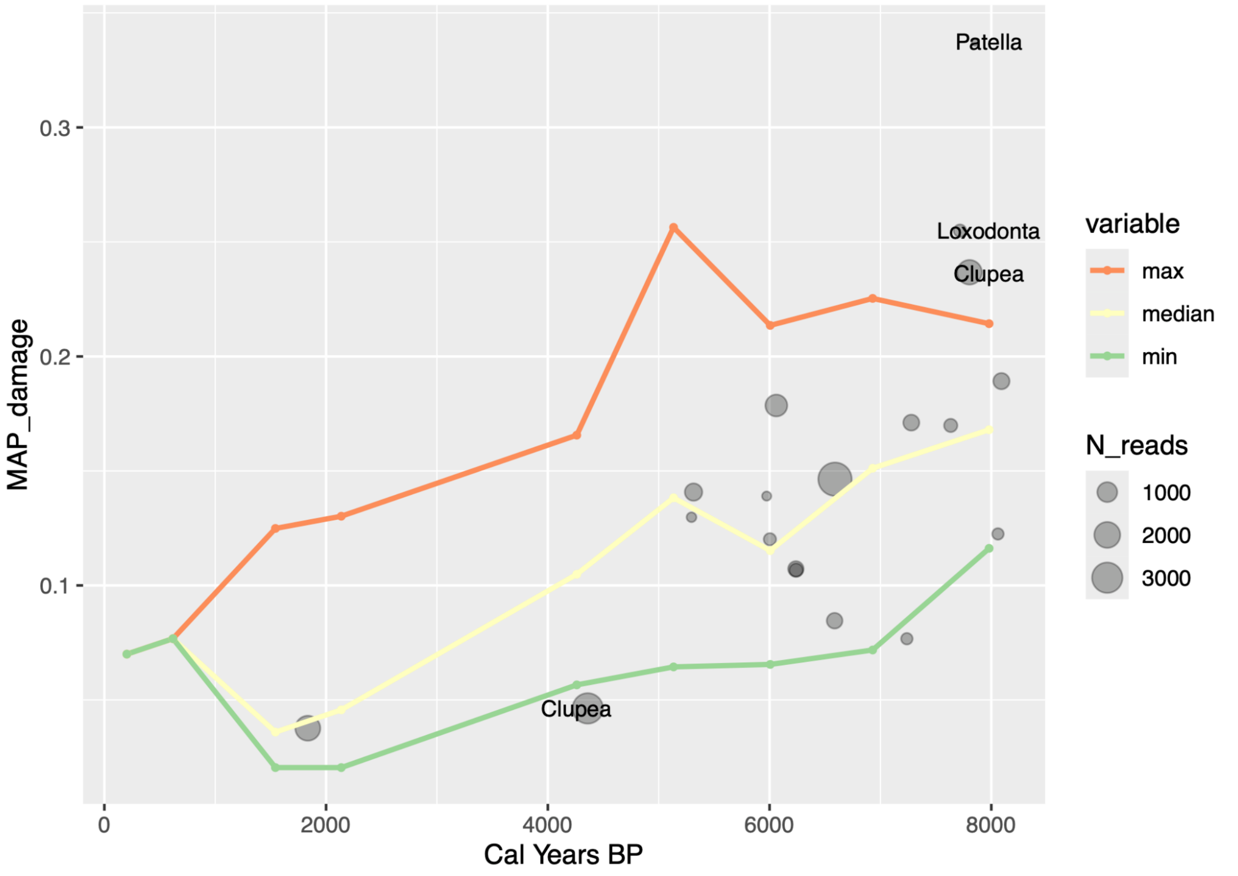


*Figure S4.1: Age-damage model showing animal reads filtered for post-mortem DNA damage. The degree of damage (measured in proportion of C -> T transitions at the terminal nucleotide position predicted by the model) is reported on the y-axis and calibrated years before the present on the X axis. The maximum, median and minimum values for all genus level observations with more than 500 reads from Viridiplantae are shown in red, yellow and green respectively. The grey bubbles show all metazoan genera observations above 99 reads with taxonomic labels applied for those outside the maximum and minimum values for Viridiplantae, bubble size indicates the number of reads per observation.*

## Supporting Information 5 - DNA damage plots

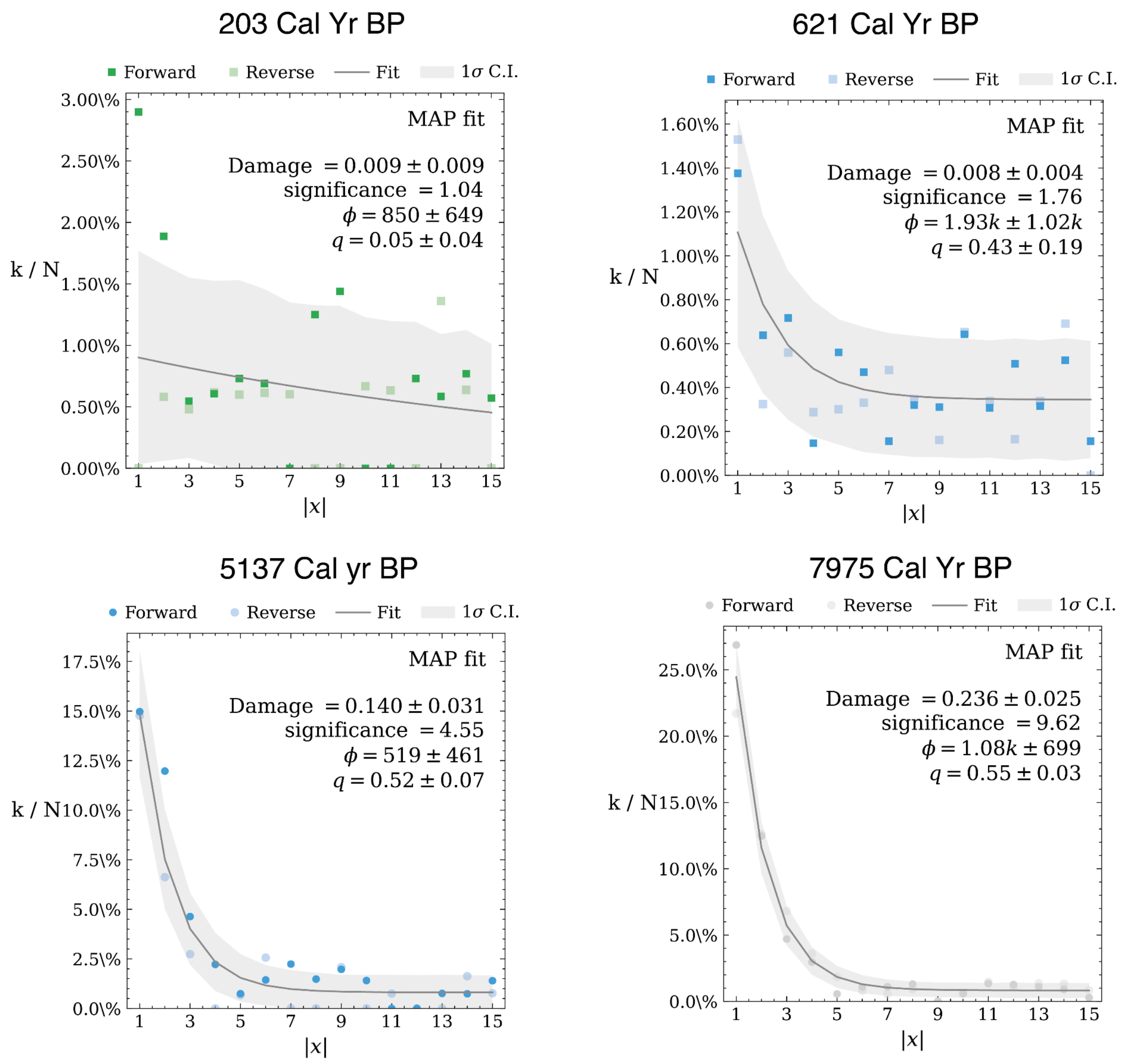


*Figure S5.1: DNA damage plots for genus Clupea from sediments with calibrated years before present shown above each figure. The position (x) along the DNA read is shown on the x-axis compared to the specific nucleotide mis-incorporations (k/N) on the forward (C > T transitions, dark-coloured dots) and reverse strand (G > A transitions, light-coloured points) on the y-axis. The uncertainty (1σ confidence interval) of the beta-binomial model fit is shown with shaded grey areas.*

**Supporting Information 6 - ASV richness estimation**

The calculation of diversity metrics from eDNA metabarcoding data is commonplace. However, it is frequently performed without expressing a series of assumed caveats

1) Different metabarcoding primers bias the observed ASV community, such that the observed ASVs from a metabarcoding experiment represent only a fraction of the target gene fragments in the eDNA pool.
2) The eDNA pool contains many possible amplifiable gene fragments, which may vary in their copy number per cell across different species.
3) Taphonomic and diagenetic processes may affect different cell types variably such that the ratios of cells are distorted after release into the environment until later capture in the environment for study during sampling.
4) The ratio of total eDNA released into the environment by different species may not reflect the biomass or count ratios of individuals as organisms release variable eDNA across their lifecycle, during different activities or under different conditions.

These caveats make it impossible to interpret the absolute number of ASVs as biologically meaningful, but since the above biases apply uniformly across samples the relative values of ASV richness can be an informative metric. With this caveat in mind, we aimed to explore if different methods for handling the metabarcoding data produced different patterns of ASV richness.
It is widely acknowledged that the proportion of positive PCR replicates to regard an ASV as a true-positive is a critical parameter to filter observation in metabarcoding (Alberdi et al., 2017). In order to test how this parameter affected our observations we created three datasets that filtered ASVs within samples to only accept ASVs in one, three or all eight PCR replicates. The results of this filtering on ASV richness is shown below. The absolute values of these three filtering approaches show large differences in the number of detected ASVs, however the overall relative pattern of ASV richness over time was consistent.

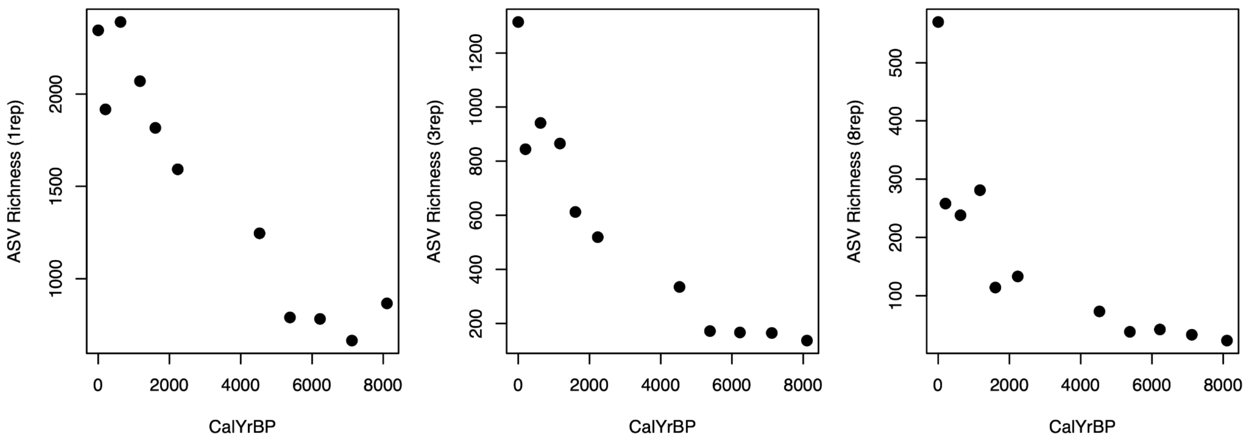


*Figure S6.1: Plots showing observed ASV richness from 18S eDNA metabarcoding of marine sediment samples. The calibrated number of years before present for each sediment sample is shown on the x-axis of each plot. The number of observed ASVs when the data is subset to include observations found in one PCR replicate (left), three or more replicates (middle) or all eight replicates (left) is shown on the y axis of each plot.*

Furthermore, we additionally tested a method that estimates richness from frequency ratios (Willis & Bunge, 2015). This approach (hereafter breakaway) produced a similar pattern to those produced by subsetting the data according to PCR replicate positive rate. The break away method showed a strong (R2=0.89-0.99) positive, significant (P<0.01) relationship with all three filtering thresholds.

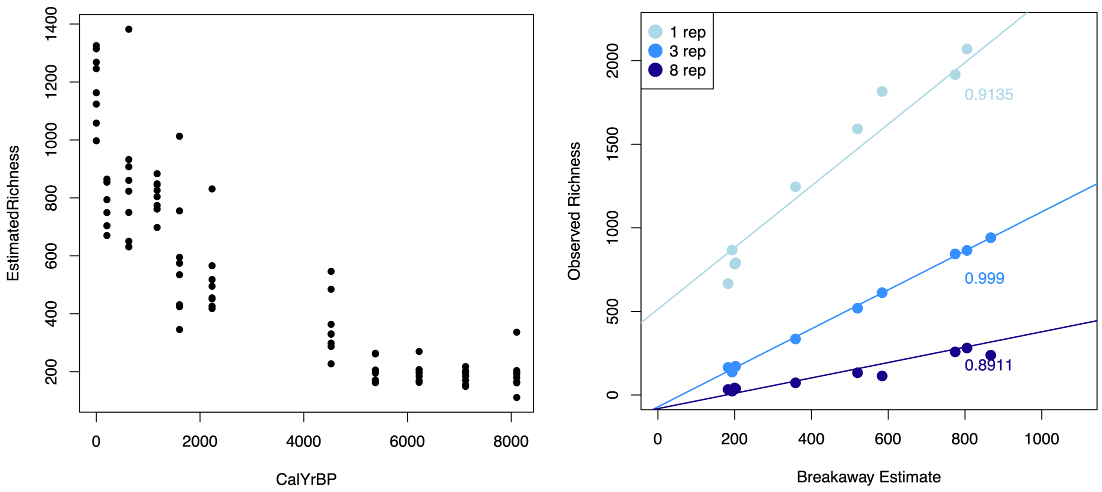


*Figure S6.2 Plots showing estimated ASV richness over time using the breakaway method (left) and a comparison of ASV richness with methods subsetting based on PCR replicates (right). The breakaway method richness estimates were calculated using the filtered 18S dataset from the main manuscript, estimating richness for each PCR replicate independently. The comparison (left) plot shows the richness observed for each of the three thresholds on the y-axis and the average breakaway richness estimate per sample on the x-axis. The three threshold datasets are coloured and a line of best fit is shown for each comparison with the R2 value for each comparison shown the corresponding colour.*

One of the key stated advantages of the breakaway method is it is tolerant to uneven effort between samples, as is standard in metabarcoding experiments given variable sequencing read depth in a typical in sequencing run. It is becoming increasingly common in eDNA metabarcoding studies to normalise the number of reads among a group of samples using rarefaction (Holman et al., 2021; Zhang et al., 2022). However, there is some discussion among microbial ecologists reading the appropriateness of rarefaction (Gloor et al., 2017; Weiss et al., 2017), with some declaring it statistically inadmissible (McMurdie & Holmes, 2014), while others show data that suggest any alternative approach critically biases diversity metrics (Schloss, 2024).

In order to test for an effect of normalisation on our richness estimates we compared the breakaway approach with cumulative sum scaling and rarefaction to normalise variable sequencing effort as described in the main manuscript. As shown below the three methods produced very similar patterns of richness across the study period.

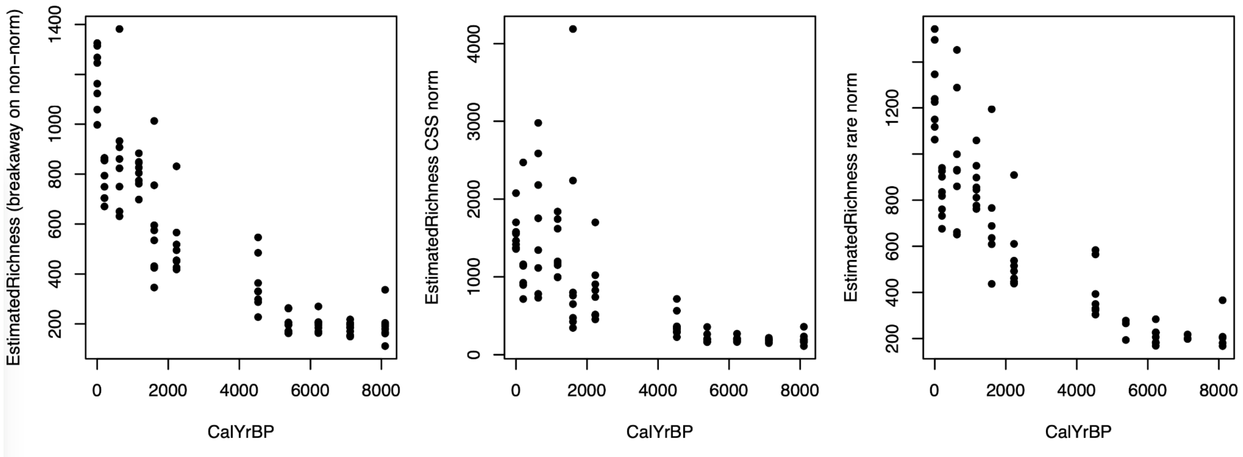


*Figure S6.3 Per replicate ASV richness over time with estimated richness on the y-axis and calibrated years before present on the x-axis for the breakaway approach (left), cumulative sum scaling normalisation (centre) and rarefaction (right).*

Overall we show that the observed ASV richness patterns are tolerant to different threatholds of PCR replicate quality filtering and normalisation method.

**Supporting Information 7 - richness comparisons**

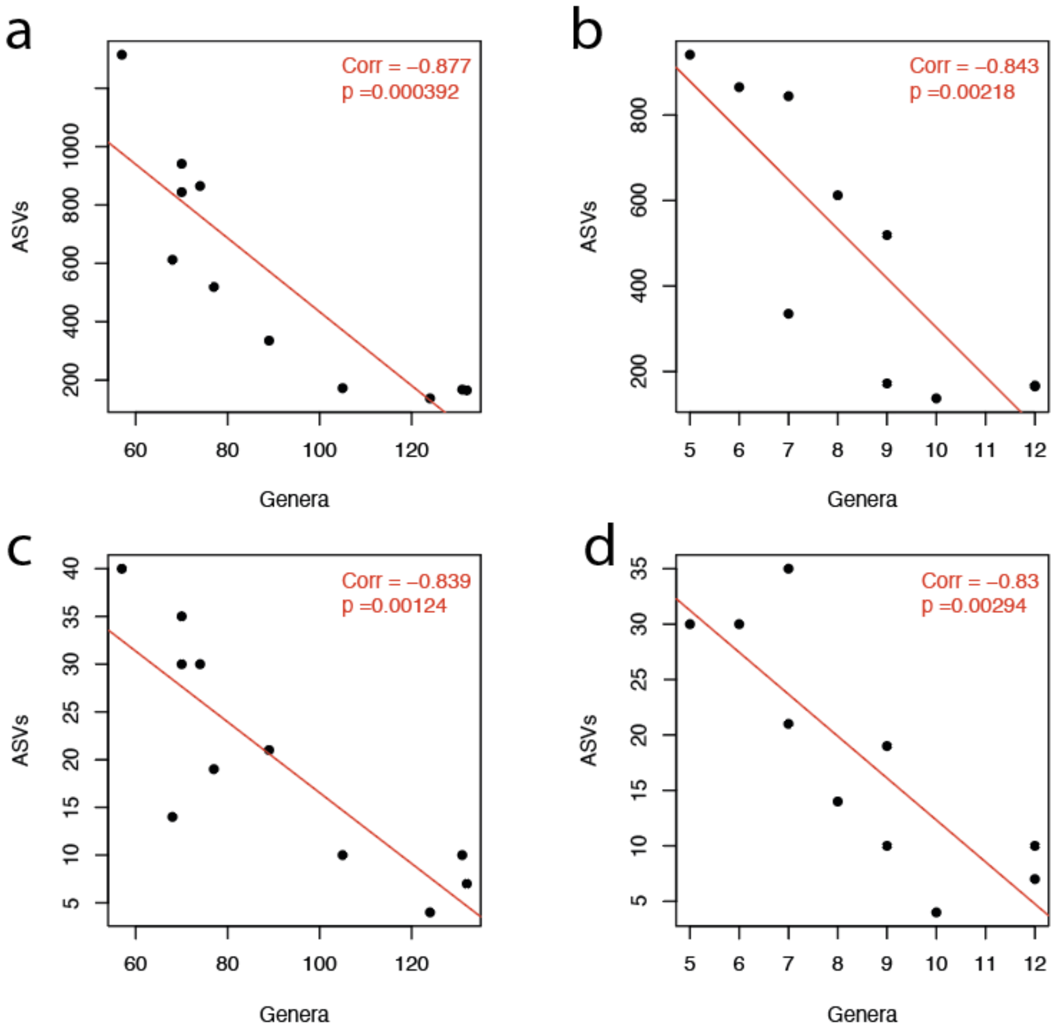


*Figure S7.1: Plots showing ASV richness from metabarcoding against detected genera from metagenomics for sedaDNA extracts from the Skagerrak. Comparisons are shown for four different datasets* ***a*** *all ASVs against all genera,* **b** *all ASVs against metazoan genera,* **c** *metazoan ASVs against all genera,* **d** *metazoan ASVs against metazoan genera. For each comparison the correlation, p value and fit of a linear regression between the variations is shown in red.*

## Supporting Information 8- beta diversity comparisons

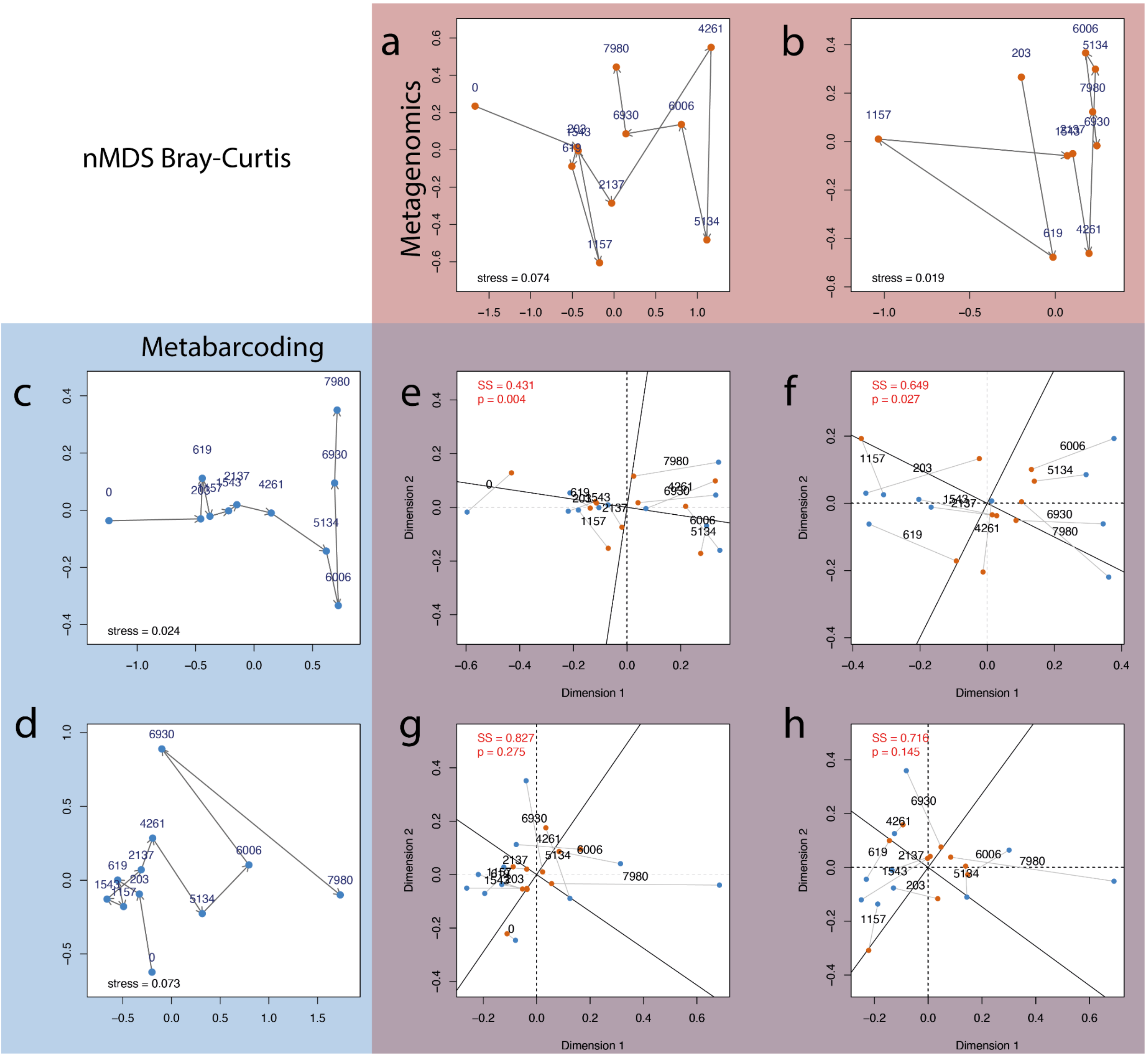


*Figure S8.1. Non-metric multidimensional scaling plots generated using Bray-Curtis dissimilarities for* ***a*** *shotgun-metagenomic detected genera including all non-viridiplantae data,* ***b*** *metazoan data,* ***c*** *all metabarcoding ASVs and* ***d*** *only metazoan ASVs. Plots* ***e-h*** *show the output of procrustes analysis with the sum of squares and p value for each test displayed in red. For each comparison blue lines indicate the relative distance between data points in each ordination and the dotted line shows the ordination rotation, both measures indicating the change from the metabarcoding to metagenomic ordination. Comparisons are shown between ordinations for* ***e*** *all genera metagenomics and all ASVs metabarcoding,* ***f*** *metazoa genera metagenomics and all ASVs metabarcoding,* ***g*** *all genera metagenomics and metazoa ASVs metabarcoding,* ***h*** *metazoa genera metagenomics and metazoa ASVs metabarcoding. For all plots the calibrated years before present is shown for each data point.*

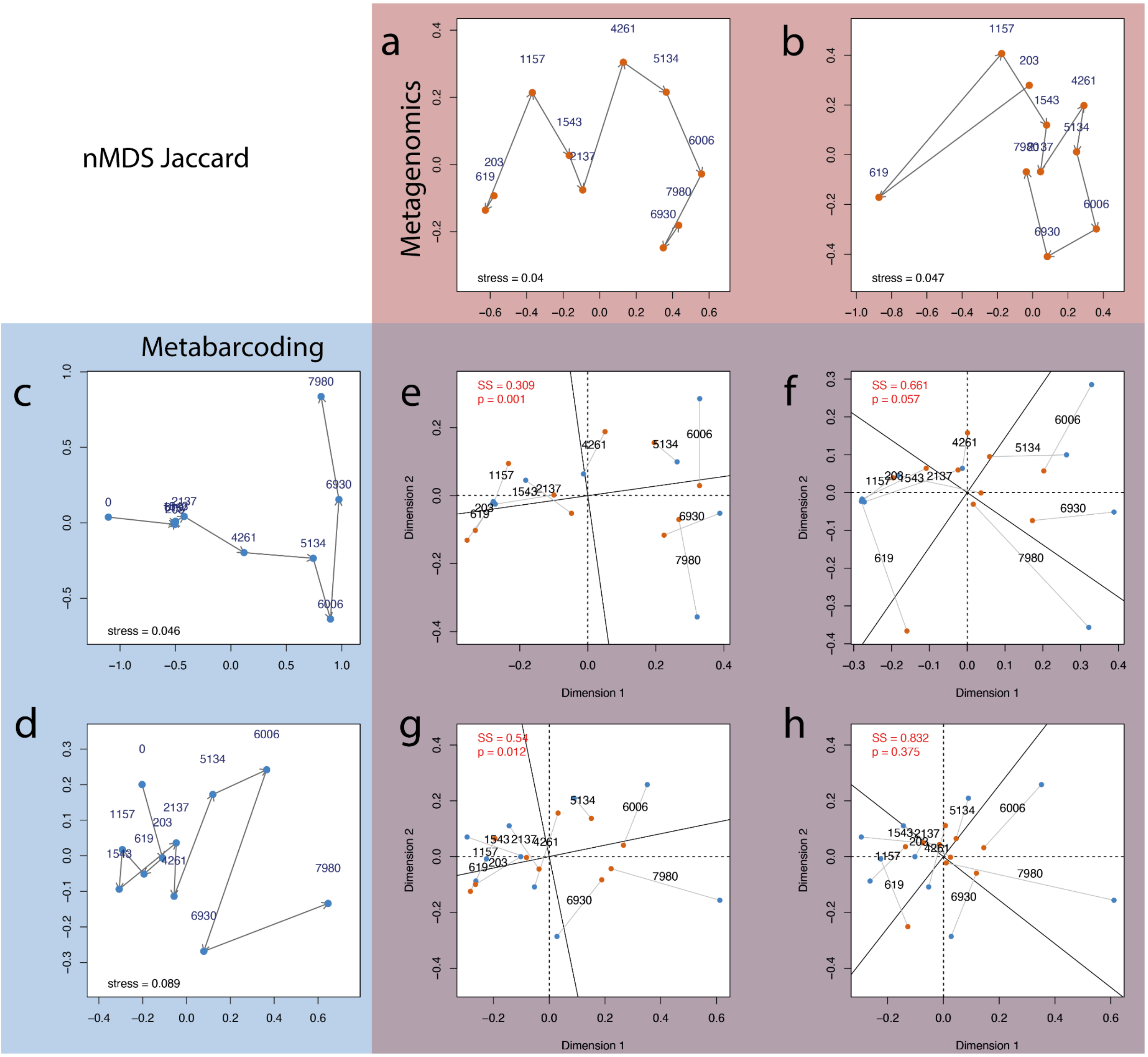


*Figure S8.2. Non-metric multidimensional scaling plots generated using Jaccard dissimilarities for **a** shotgun-metagenomic detected genera including all non-viridiplantae data, **b** metazoan data, **c** all metabarcoding ASVs and **d** only metazoan ASVs. Plots **e-h** show the output of procrustes analysis with the sum of squares and p value for each test displayed in red. For each comparison blue lines indicate the relative distance between data points in each ordination and the dotted line shows the ordination rotation, both measures indicating the change from the metabarcoding to metagenomic ordination. Comparisons are shown between ordinations for **e** all genera metagenomics and all ASVs metabarcoding, **f** metazoa genera metagenomics and all ASVs metabarcoding, **g** all genera metagenomics and metazoa ASVs metabarcoding, **h** metazoa genera metagenomics and metazoa ASVs metabarcoding. For all plots the calibrated years before present is shown for each data point.*

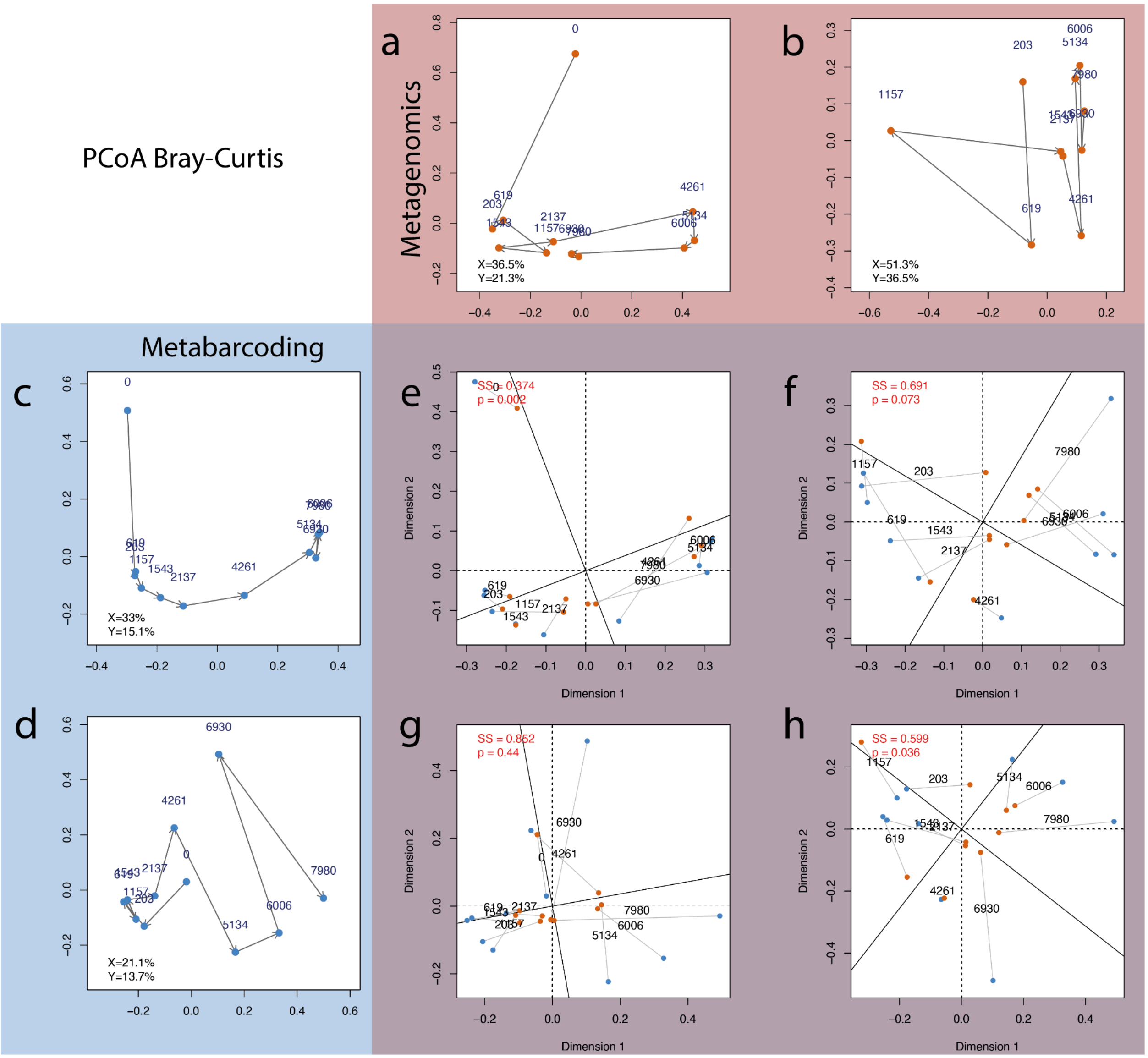


*Figure S8.3. Multidimensional scaling plots generated using Bray-Curtis dissimilarities for **a** shotgun-metagenomic detected genera including all non-viridiplantae data, **b** metazoan data, **c** all metabarcoding ASVs and **d** only metazoan ASVs. Plots **e-h** show the output of procrustes analysis with the sum of squares and p value for each test displayed in red. For each comparison blue lines indicate the relative distance between data points in each ordination and the dotted line shows the ordination rotation, both measures indicating the change from the metabarcoding to metagenomic ordination. Comparisons are shown between ordinations for **e** all genera metagenomics and all ASVs metabarcoding, **f** metazoa genera metagenomics and all ASVs metabarcoding, **g** all genera metagenomics and metazoa ASVs metabarcoding, **h** metazoa genera metagenomics and metazoa ASVs metabarcoding. For all plots the calibrated years before present is shown for each data point.*

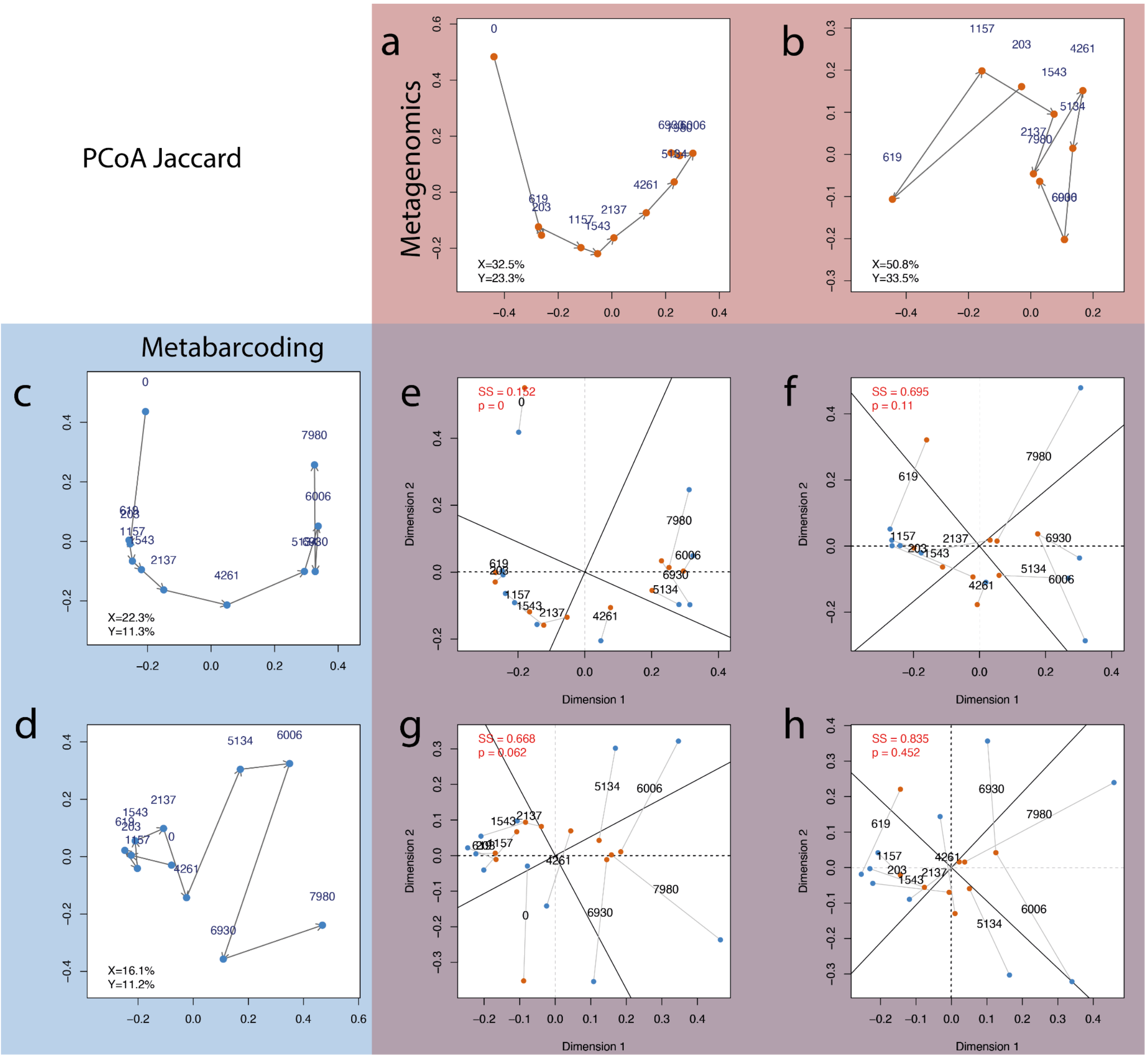


*Figure S8.4 .Multidimensional scaling plots generated using Jaccard dissimilarities for* ***a*** *shotgun-metagenomic detected genera including all non-viridiplantae data,* ***b*** *metazoan data,* ***c*** *all metabarcoding ASVs and* ***d*** *only metazoan ASVs. Plots* ***e-h*** *show the output of procrustes analysis with the sum of squares and p value for each test displayed in red. For each comparison blue lines indicate the relative distance between data points in each ordination and the dotted line shows the ordination rotation, both measures indicating the change from the metabarcoding to metagenomic ordination. Comparisons are shown between ordinations for* ***e*** *all genera metagenomics and all ASVs metabarcoding,* ***f*** *metazoa genera metagenomics and all ASVs metabarcoding,* ***g*** *all genera metagenomics and metazoa ASVs metabarcoding,* ***h*** *metazoa genera metagenomics and metazoa ASVs metabarcoding. For all plots the calibrated years before present is shown for each data point.*

## Supporting Information 9 - Mantel test outputs

*Table S9.1: Table with test statistics (r) and p values for Mantel tests between dissimilarity matrices generated from metabarcoding and metagenomic analyses.*

| **Bray-Curtis Dissimilarity** | | **Metagenomics** | |
|---|---|---|---|
| | | All Data | Metazoa |
| **Metabarcoding** | All Data | r=0.631,p=0.0009 | r=0.045,p=0.3881 |
| | Metazoa | r=0.337,p=0.0716 | r=-0.094,p=0.5818 |

**Jaccard Index**

| | | **Metagenomics** | |
|---|---|---|---|
| | | All Data | Metazoa |
| **Metabarcoding** | All Data | r=0.523,p=0.0004 | r=0.172,p=0.2374 |
| | Metazoa | r=0.3,p=0.1179 | r=0.029,p=0.4294 |

**Supporting Information 10 - Observed length distribution of sequences and effect on diversity**

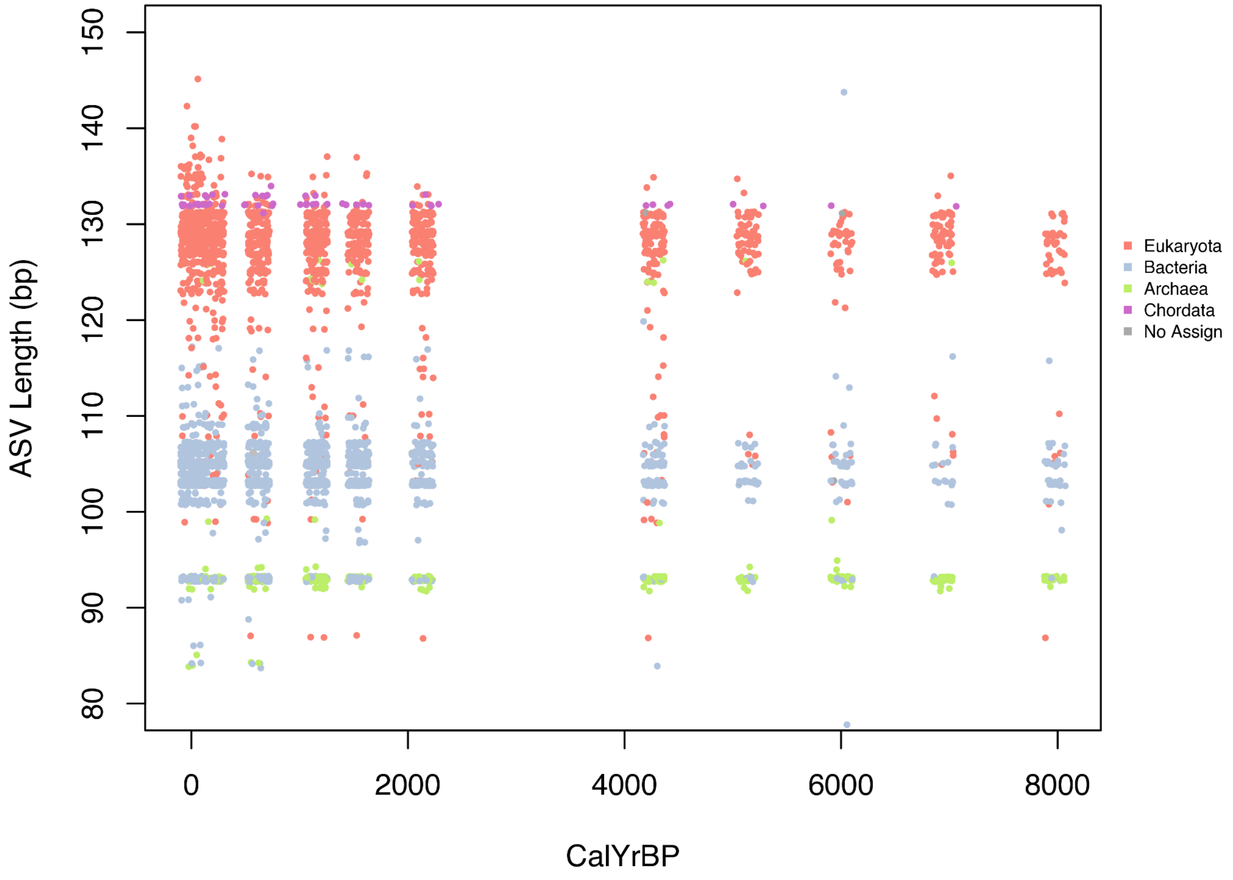


*Figure S10.1 Length of ASVs (base-pairs) detected over time (calibrated years before present) from 18S eDNA metabarcoding of marine sediment samples. Point colour indicates taxonomic assignment for a given detection*

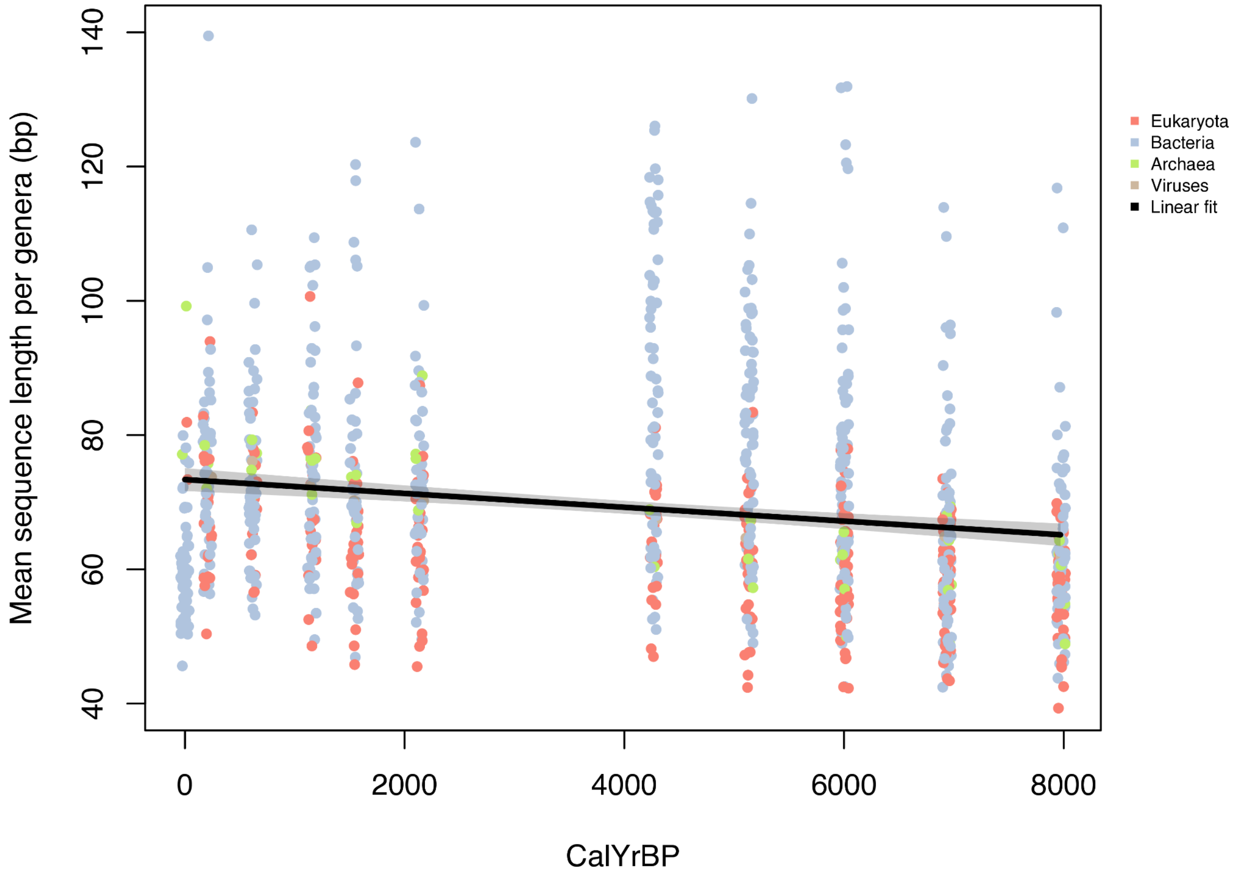


*Figure S10.2 Average length (base-pairs) of sequences per metagenomic genera (base-pairs) detected over time (calibrated years before present) from shot-gun metagenomic sequencing of marine sediment samples. Point colour indicates taxonomic assignment for a given detection. A predicted fit from a significant linear regression ($\alpha = 0.01$) is shown in black with 95% confidence intervals shaded grey.*

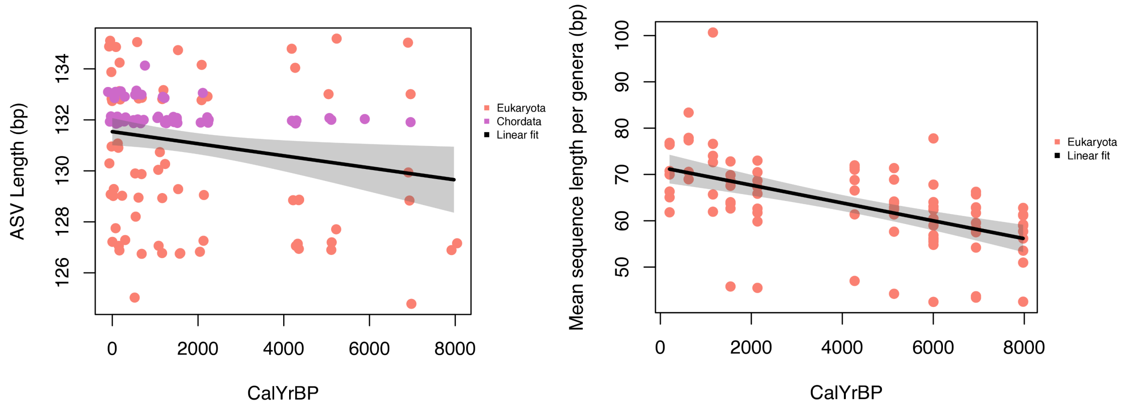


*Figure S10.3 Length of metazoa ASVs (base-pairs) detected (left) and Average length (base-pairs) of sequences per metagenomic metazoa genera (right) over time (calibrated years before present) from 18S eDNA metabarcoding and shotgun-metagenomic sequencing of marine sediment samples. Point colour indicates taxonomic assignment for a given detection. A predicted fit from a significant linear regression ($\alpha$ = 0.01) is shown in black with 95% confidence intervals shaded grey.*

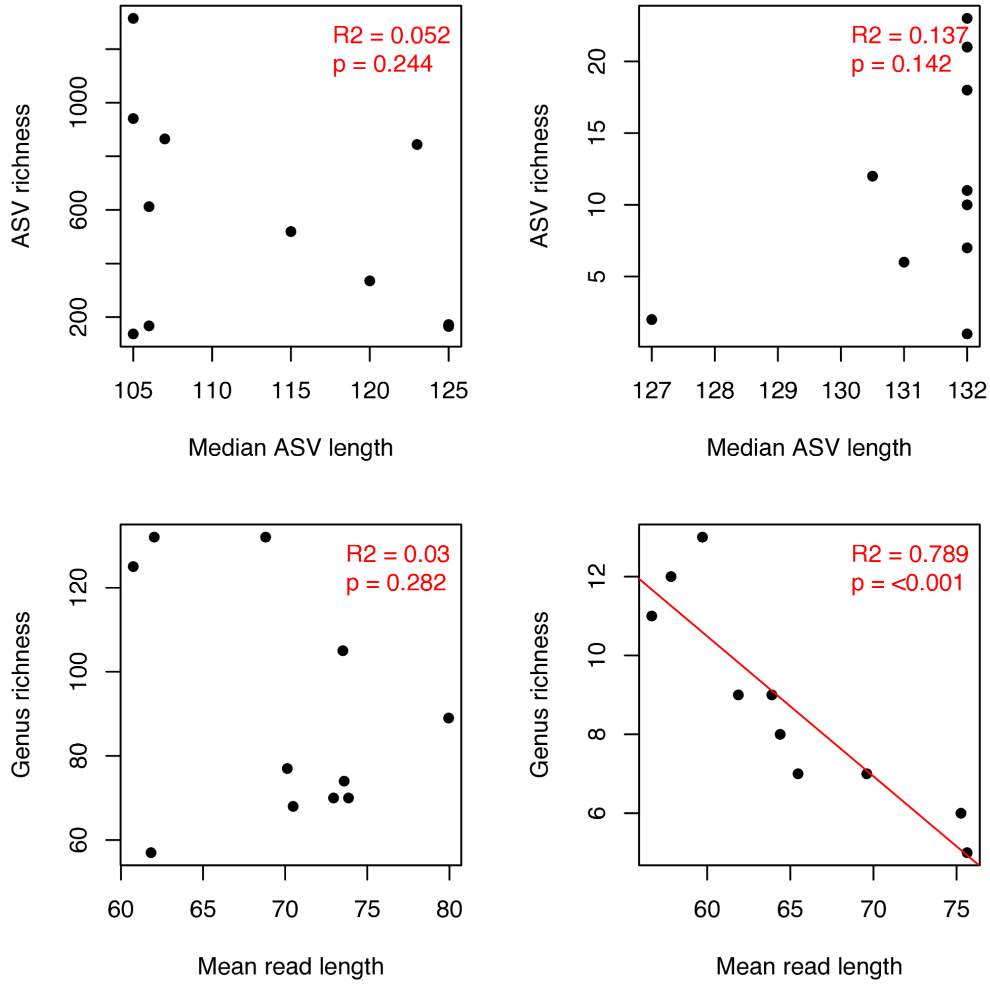


*Figure S10.4 Taxonomic richness compared to detected sequencing length for metabarcoding of all ASVs (top left), metazoa only ASVs (top right), all metagenomic taxa (bottom left) and metazoa metagenomic taxa only (bottom right). For all comparisons the R squared and p value for a linear regression is shown with a best fit line shown in red for significant (α = 0.01) relationships.*

To test the effect of sequence length on beta diversity the four datasets (metabarcoding all, metabarcoding metazoans, metagenomics all, metabarcoding all) analysed in the main manuscript were analysed as follows. Two distance-based redundancy analyses were conducted using Jaccard and Bray-curtis dissimilarities, with each redundancy analysis variation in the community distance matrix was explained using the sequence length metrics shown above (Figure S10.1-10.3). In all eight analyses (four datasets by two distance metrics) the length metric did not explain a significant proportion of the variance in the distance matrix (α = 0.01).

## Supporting Information 11 - Metabarcoding replicate ordinations

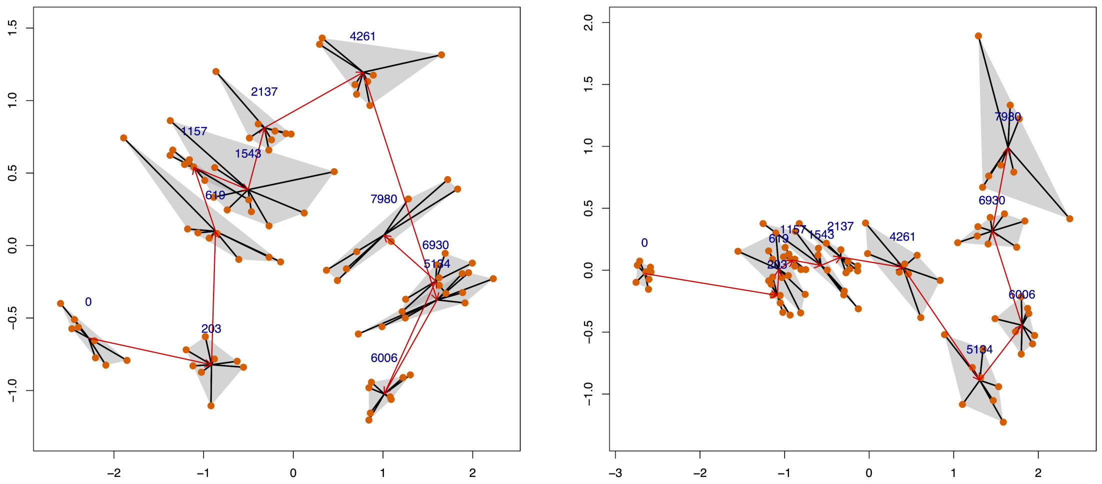


*Figure S11.1 Non-metric multidimensional scaling plots generated using Bray-Curtis (left) and Jaccard (right) dissimilarities from marine sediment metabarcoding. Individual replicates are marked with orange points with a convex hull around the replicates per each sample shown in grey. The distance to the centroid for replicates from each sample is shown with black lines. The sample date as presented in the main manuscript is presented in calibrated years before present and shown in blue. Sample progression through time is shown by a red line connecting sample replicate centroids.*

**Supporting Information 12 - Fungal detections in sediment core**

During subsampling the surface of the stored core displayed evidence of fungal growth during storage (see Figure S12.1 below). In line with typical methods for subsampling from sediment cores for sedaDNA analysis, all surface sediment was removed and only inner material processed further. During this sampling there was no visible evidence of fungal growth below the surface of the core.

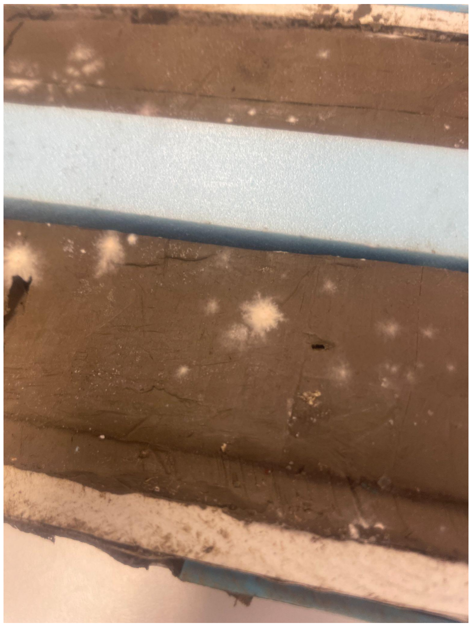

*Figure S12.1: Photograph showing white fungal growth on the surface of the split core. The blue polystyrene spacer is from previous subsampling.*

Following these observations during subsampling there was a large proportion of the relative reads in the 18S metabarcoding dataset assigned to a single ASV in the class *Eurotiomycetes* as seen in Fig. S10.2 below. This ASV had up to 60% relative read abundance in some replicates (e.g. a replicate from the sample collected at 2050cm), and above 30% in all replicates from samples in 2050cm - 2450cm. A blastn search against the entire NCBI nt database on Feb 12th 2024 showed a 100% coverage 100% identity match to species from >10 fungal genera. It was not possible to make an assignment of this ASV to a likely genera, likely reflecting the lack of resolution in the metabarcoding marker to delineate fungal taxa. We chose not to exclude this taxa from the statistical tests as it is a true positive-detection in the material and highlights potential issues with using older material.

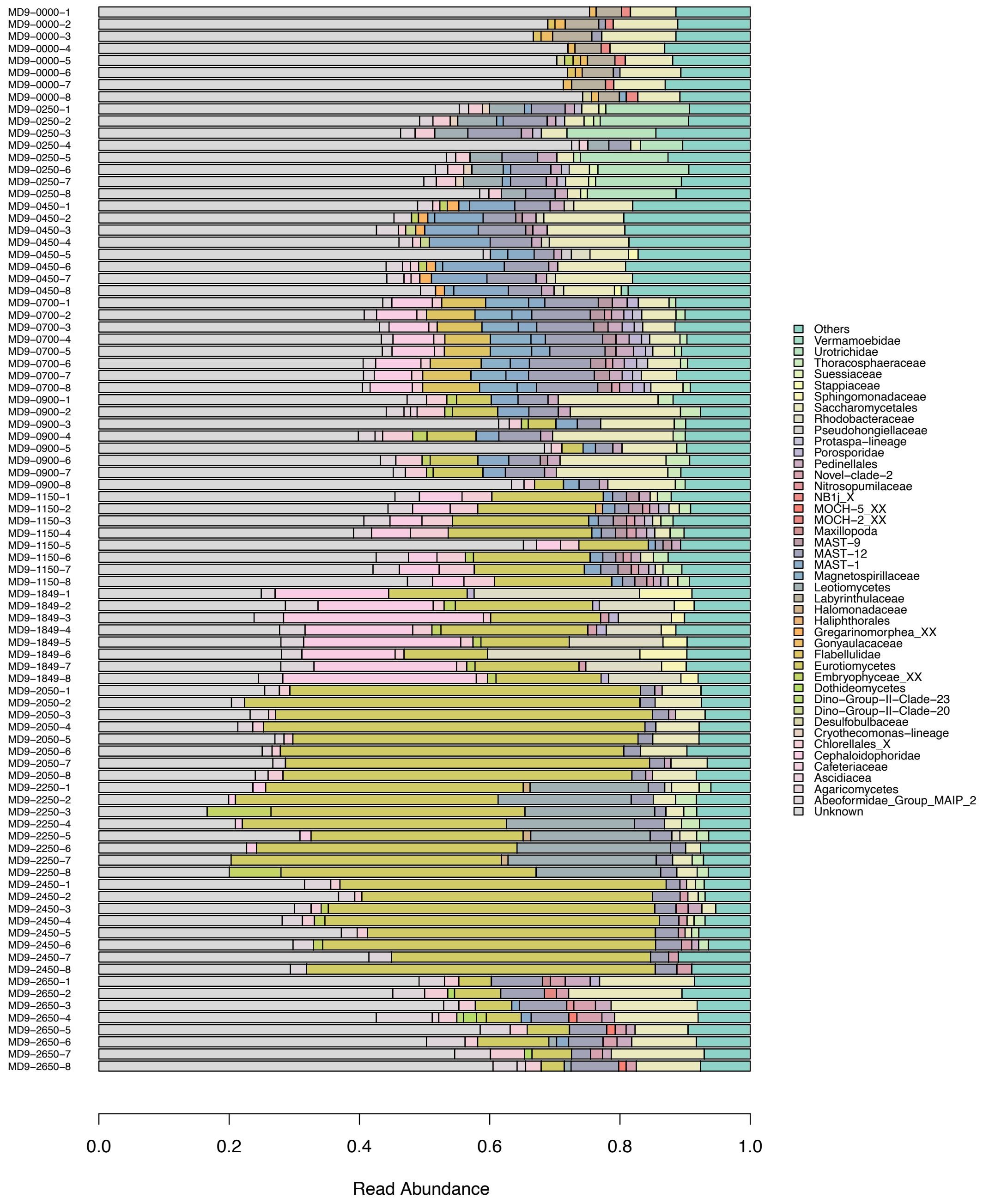


*Figure S12.2 (top) Relative read abundance of ASVs assigned to different taxonomic families across replicate samples from 18S metabarcoding of marine sediments. Taxa represented by <1% of reads per sample are shown in the category 'other'. (bottom) Relative ASV counts of ASVs assigned to different taxonomic classes across replicate samples from 18S metabarcoding of marine sediments. Taxa represented by <1% of reads per sample are shown in the category 'other'.*

## Supporting References


*Alberdi, A., Aizpurua, O., Gilbert, M. T. P., & Bohmann, K. (2017). Scrutinizing key steps for reliable metabarcoding of environmental samples. Methods in Ecology and Evolution, 9(1), 134–147.*

*European Environmental Agency. (2012). European catchments and Rivers network system (Ecrins) [dataset]. https://www.eea.europa.eu/en/datahub/datahubitem-view/a9844d0c-6dfb-4c0c-a693-7d991cc82e6e*

*Froese, R., & Pauly, D. (2024). FishBase: A Global Information System on Fishes. www.fishbase.org*

*GEBCO Bathymetric Compilation Group. (2023). The GEBCO_2023 Grid - a continuous terrain model of the global oceans and land [dataset]. NERC EDS British Oceanographic Data Centre NOC. https://doi.org/10.5285/F98B053B-0CBC-6C23-E053-6C86ABC0AF7B*

*Gloor, G. B., Macklaim, J. M., Pawlowsky-Glahn, V., & Egozcue, J. J. (2017). Microbiome Datasets Are Compositional: And This Is Not Optional. Frontiers in Microbiology, 8, 2224.*

*Gyllencreutz, R., Backman, J., Jakobsson, M., Kissel, C., & Arnold, E. (2006). Postglacial palaeoceanography in the Skagerrak. Holocene, 16(7), 975–985.*

*Holman, L. E., de Bruyn, M., Creer, S., Carvalho, G., Robidart, J., & Rius, M. (2021). Animals, protists and bacteria share marine biogeographic patterns. Nature Ecology & Evolution, 5(6), 738–746.*

*Jensen, T. Z. T., Niemann, J., Iversen, K. H., Fotakis, A. K., Gopalakrishnan, S., Vågene, Å. J., Pedersen, M. W., Sinding, M.-H. S., Ellegaard, M. R., Allentoft, M. E., Lanigan, L. T., Taurozzi, A. J., Nielsen, S. H., Dee, M. W., Mortensen, M. N., Christensen, M. C., Sørensen, S. A., Collins, M. J., Gilbert, M. T. P., … Schroeder, H. (2019). A 5700 year-old human genome and oral microbiome from chewed birch pitch. Nature Communications, 10(1), 5520.*

*Key, F. M., Posth, C., Krause, J., Herbig, A., & Bos, K. I. (2017). Mining Metagenomic Data Sets for Ancient DNA: Recommended Protocols for Authentication. Trends in Genetics: TIG, 33(8), 508–520.*

*Kjær, K. H., Winther Pedersen, M., De Sanctis, B., De Cahsan, B., Korneliussen, T. S., Michelsen, C. S.,*

*Sand, K. K., Jelavić, S., Ruter, A. H., Schmidt, A. M. A., Kjeldsen, K. K., Tesakov, A. S., Snowball, I., Gosse, J. C., Alsos, I. G., Wang, Y., Dockter, C., Rasmussen, M., Jørgensen, M. E., … Willerslev, E. (2022). A 2-million-year-old ecosystem in Greenland uncovered by environmental DNA. Nature, 612(7939), 283–291.*

*Laine, J., Mak, S. S. T., Martins, N. F. G., Chen, X., Gilbert, M. T. P., Jones, F. C., Pedersen, M. W., Romundset, A., & Foote, A. D. (2024). Late Pleistocene stickleback environmental genomes reveal the chronology of freshwater adaptation. Current Biology: CB, 34(5), 1142–1147.e6.*

*Latz, M. A. C., Grujcic, V., Brugel, S., Lycken, J., John, U., Karlson, B., Andersson, A., & Andersson, A. F. (2022). Short- and long-read metabarcoding of the eukaryotic rRNA operon: Evaluation of primers and comparison to shotgun metagenomics sequencing. Molecular Ecology Resources, 22(6), 2304–2318.*

*Llamas, B., Valverde, G., Fehren-Schmitz, L., Weyrich, L. S., Cooper, A., & Haak, W. (2017). From the field to the laboratory: Controlling DNA contamination in human ancient DNA research in the high-throughput sequencing era. STAR: Science & Technology of Archaeological Research, 3(1), 1–14.*

*McMurdie, P. J., & Holmes, S. (2014). Waste not, want not: why rarefying microbiome data is inadmissible. PLoS Computational Biology, 10(4), e1003531.*

*Sandoval-Velasco, M., Dudchenko, O., Rodríguez, J. A., Estrada, C. P., Dehasque, M., Fontsere, C., Mak, S. S. T., Plotnikov, V., Khan, R., Weisz, D., Contessoto, V. G., Oliveira Junior, A. B., Kalluchi, A., Omer, A. D., Batra, S. S., Shamim, M. S., Durand, N. C., O'Connell, B., Roca, A. L., … Aiden, E. L. (2023). Three-dimensional genome architecture persists in a 52,000-year-old woolly mammoth skin sample. In bioRxiv (p. 2023.06.30.547175). https://doi.org/10.1101/2023.06.30.547175*

*Schloss, P. D. (2024). Rarefaction is currently the best approach to control for uneven sequencing effort in amplicon sequence analyses. mSphere, e0035423.*

*Ukkonen, P., Aaris-Sørensen, K., Arppe, L., Clark, P. U., Daugnora, L., Lister, A. M., Lõugas, L., Seppä, H., Sommer, R. S., Stuart, A. J., Wojtal, P., & Zupiņš, I. (2011). Woolly mammoth (Mammuthus*

*primigenius Blum.) and its environment in northern Europe during the last glaciation. Quaternary Science Reviews, 30(5), 693–712.*

*Weiss, S., Xu, Z. Z., Peddada, S., Amir, A., Bittinger, K., Gonzalez, A., Lozupone, C., Zaneveld, J. R., Vázquez-Baeza, Y., Birmingham, A., Hyde, E. R., & Knight, R. (2017). Normalization and microbial differential abundance strategies depend upon data characteristics. Microbiome, 5(1), 27.*

*Whitmore, L., McCauley, M., Farrell, J. A., Stammnitz, M. R., Koda, S. A., Mashkour, N., Summers, V., Osborne, T., Whilde, J., & Duffy, D. J. (2023). Inadvertent human genomic bycatch and intentional capture raise beneficial applications and ethical concerns with environmental DNA. Nature Ecology & Evolution, 7(6), 873–888.*

*Willis, A., & Bunge, J. (2015). Estimating diversity via frequency ratios. Biometrics, 71(4), 1042–1049.*

*Zhang, S., Zheng, Y., Zhan, A., Dong, C., Zhao, J., & Yao, M. (2022). Environmental DNA captures native and non-native fish community variations across the lentic and lotic systems of a megacity. Science Advances, 8(6), eabk0097.*
